# Keystone roles of carbon-degrading enzyme activities in mediating carbon in soils subjected to straw return: a global meta-analysis

**DOI:** 10.3389/fmicb.2026.1739110

**Published:** 2026-02-04

**Authors:** Somdee Somchanh, Yue Li, Ling Yang, Ran Wei, Yuxuan Zhang, Bing Liu, Qingwen Jiang, Qiliang Yang

**Affiliations:** 1 Faculty of Environmental Science and Engineering, Kunming University of Science and Technology, Kunming, China; 2 Faculty of Modern Agricultural Engineering, Kunming University of Science and Technology, Kunming, China; 3 Yunnan Key Laboratory of Efficient Utilization of Agricultural Water Resources and Intelligent Control, Faculty of Modern Agricultural Engineering, Kunming University of Science and Technology, Kunming, China; 4 Yunnan International Joint Laboratory of Intelligent Agricultural Engineering Technology and Equipment, Faculty of Modern Agricultural Engineering, Kunming University of Science and Technology, Kunming, China; 5 Faculty of Information Engineering and Automation, Kunming University of Science and Technology, Kunming, China; 6 Faculty of Science, Kunming University of Science and Technology, Kunming, China

**Keywords:** extracellular enzyme activities, meta-analysis, microbial biomass carbon, soil organic carbon, straw return

## Abstract

**Introduction:**

Straw return exerts a profound impact on soil fertility, with particularly critical implications for soil carbon (C) pools. Soil hydrolytic C-degrading extracellular enzyme activities (Hy-EEAs) play a central role in soil C cycling. However, the effects of straw return on Hy-EEAs, below-ground C dynamics, and the underlying regulatory mechanisms have not been fully elucidated.

**Methods:**

In this study, we evaluated the effects of straw incorporation on Hy-EEAs and below-ground C, as well as their potential relationships, by synthesizing 211 observations from 68 published field studies worldwide.

**Results:**

On average, straw return significantly enhanced Hy-EEAs by 25% but had no effect on β-xylosidase. Straw return significantly increased dissolved organic carbon, easily oxidizable carbon, light fraction organic carbon, particulate organic carbon, microbial biomass carbon, and soil organic carbon by 27, 24, 51, 34, 31, and 20%, respectively, compared to the no-straw-return treatment. The effect of straw return on Hy-EEAs decreased with increasing experiment duration (≥ 10 years). Straw return effects on Hy-EEAs increased with the incorporation of straw. The response ratios (*lnR*) of microbial biomass C content and soil organic carbon (SOC) storage to straw return were positively correlated with the *lnR* of Hy-EEAs; however, no clear relationships were found between the *lnR* of soil dissolved organic C (DOC), easily oxidizable C (EOC), light fraction organic C (LFOC), and particulate organic C (POC) and the *lnR* of Hy-EEAs.

**Discussion:**

These results suggest that straw return stimulation of Hy-EEAs exhibited a key role in regulating below-ground C dynamics. Future biogeochemistry models could incorporate the observed relationships in this study between the soil C pool and Hy-EEAs, which can improve model predictions of C in soils under straw return in agricultural systems.

## Highlights

- Straw return increased hydrolytic carbon-acquiring enzyme activities (Hy-EEAs).
- Straw return stimulation of Hy-EEAs was positively correlated with the responses of microbial biomass carbon.
- Straw return increased soil organic carbon storage, which was positively associated with Hy-EEAs.
- Future model projections could consider the above relationships for cropping systems.

## 1 Introduction

Soils store three to four times as much carbon (C) as the atmosphere (Tarnocai et al., 2009; Terrer et al., 2016; Lal, 2004). Changes in soil C in response to agricultural practices (e.g., straw return) will have cascading impacts on future climate change (Trumbore et al., 1996). Crop straw production exceeds five billion tons per year globally (Cherubin et al., 2018), and a large proportion of crop straw is returned again to agricultural lands (Xia et al., 2018). Considerable studies have demonstrated that straw return can help improve soil enzyme activities and soil C cycling (Davidson and Janssens, 2006; Lal, 2005; Liu et al., 2014; Majumder et al., 2008; Powlson et al., 2008; Singh et al., 2008). Specifically, straw acts as a direct organic C substrate to increase soil organic carbon (SOC) fractions (e.g., dissolved organic carbon and microbial biomass carbon) and stimulate the activity of C-cycling enzymes (e.g., *β*-glucosidase and cellulase). These changes further accelerate the decomposition of organic matter, promote C sequestration in soil aggregates, and enhance the turnover of active C pools, thereby regulating the overall soil C cycling process. Therefore, it is critical to understand how straw return affects soil enzyme activities (Lu et al., 2009; Shang et al., 2011; Stewart et al., 2007) and potential mechanisms associated with C in arable soils.

Soil extracellular enzyme activities (EEAs) are keystone indicators of microbial activities linked to substrate dynamics (Burns et al., 2013; Sinsabaugh and Findlay, 1995; Sinsabaugh et al., 2008). The EEAs are therefore believed to be proximate agents and the rate-limiting step in soil C decomposition (Bardgett et al., 2008; Davidson and Janssens, 2006). Straw return supplies labile organic substrates to soil microbes, thereby enhancing microbial activity and biomass; this, in turn, accelerates the turnover of soil organic carbon pools and modulates the accumulation of soil total carbon content (Sharma et al., 2019; Zhang et al., 2017; Zhao S. et al., 2016). For example, Bray et al. (2012) and Yang et al. (2012) stated that straw return can affect microbial physiology and community and the amount of organic substrates, changing the microbial production of soil EEAs (Li et al., 2017; Tiemann and Billings, 2011). Soil EEAs can decompose substrates of varying complexity and composition (Liu et al., 2014; Sinsabaugh and Shah, 2012); therefore, knowledge of how they respond to straw return may help to develop strategies for enhancing soil C stocks.

Soil EEAs associated with microbial C degradation (i.e., a group of hydrolytic enzymes that soil microbes produce to decompose polysaccharides) include *α*-1,4-glucosidase (AG), *β*-1,4-xylosidase (BX), *β*-1,4-glucosidase (BG), and β-d-cellobiosidase (CBH) (Deng and Tabatabai, 1995; Jian et al., 2016). The responses of soil hydrolytic C-degrading EEAs (Hy-EEAs) under straw return have been explored for decades, varying in magnitude and direction across many studies (Huang et al., 2021; Jarvis et al., 1996; Stemmer et al., 1999; Wingeyer et al., 2012). Research has shown that straw return can increase (Bhattacharyya et al., 2012; Hazarika et al., 2009; Li et al., 2020; Li Y. et al., 2022), decrease (Liang et al., 2018), or have no effect (Dai et al., 2022; Sun et al., 2021) on Hy-EEAs in cropping ecosystems. Although the wealth of studies has evaluated straw return stimulation of individual Hy-EEAs (Burns et al., 2013; Wallenstein and Burns, 2011), it is still unclear which specific enzyme is significantly affected by straw return and how Hy-EEA’s responses to straw return affect below-ground C dynamics. Moreover, the relative contribution of environmental variables in driving the responses of Hy-EEAs and C in soils to straw return is elusive. These uncertainties and knowledge gaps impede further understanding of Hy-EEAs and below-ground C dynamics, as well as their associations under straw return.

Straw return is a widely adopted agricultural practice for enhancing soil fertility and mitigating climate change via carbon (C) sequestration. Previous studies have extensively documented the significant effects of straw return on soil enzyme activities and soil C cycling, but these findings are highly variable across different soil types, climate conditions, and straw management strategies. Notably, a majority of existing studies focus on individual hydrolytic extracellular enzyme activities (Hy-EEAs) or soil C fractions in isolation, and few studies have explored the mechanistic linkages between Hy-EEAs and below-ground C dynamics. This disconnect hinders a comprehensive understanding of how straw return regulates soil C cycling processes at a global scale. Moreover, conventional meta-analyses often overlook non-linear relationships and the relative importance of multiple driving factors, limiting the robustness of their conclusions. So far, however, there is no systematic assessment of the responses of Hy-EEAs to straw return and whether these responses can be related to below-ground C dynamics. A meta-analysis combined with the advanced model selection was therefore conducted to synthesize the effects of straw return on Hy-EEAs and C in soils and potential association between them. A random-meta-forest approach was used to account for multiple drivers simultaneously, such as non-linear relationships. We predicted that differential responses of C in soils and Hy-EEAs to straw return depend on soil and climate factors and straw management. We also hypothesized that there were potential linkages between changes in C in soils and straw return stimulation of Hy-EEAs. Therefore, the objectives of this study were (1) to understand how Hy-EEAs and C in soils respond to straw return, (2) to identify the key soil and climate predictors of Hy-EEAs and soil C pool associated with straw return and rank their importance, and (3) to discuss the possible implications for below-ground C dynamics.

## 2 Materials and methods

### 2.1 Data collection

We searched Web of Science^1^ and China National Knowledge Infrastructure (CNKI)^2^ databases for peer-reviewed articles. The literature search followed the procedure of Preferred Reporting Items for Systematic Reviews and Meta-Analyses (PRISMA; Moher et al., 2009) (Supplementary Figure S1). Search terms were either “straw,” “straw incorporation,” “straw mulching,” “straw return,” “crop residue,” or “stover,” and either “enzyme,” “hydrolytic enzyme,” “glucosidase,” “*β*-1,4-glucosidase,” “*α*-1,4-glucosidase,” “EEA,” “cellulase,” β-1,4-D-cellobiohydrolase,” “soil carbon,” “soil microbial biomass,” “soil organic carbon,” or “β-1,4-xylosidase.” The dataset was established based on the following criteria: (1) the initial climatic conditions and soil physicochemical characteristics were same between no-straw-return and straw return treatments; (2) experimental duration must be clear, with field experiments (not surveys or pot experiments) lasting at least 1 year; (3) if an article contained results from multiple soil depths, we used data from the uppermost soil layer. Moreover, if any study contained duplicate results in different growing years for the same experiment, we included only the latest sampling time in this analysis; and (4) the experiment location was stated. For each article, we extracted mean values (*Mean*), replicate numbers (*n*), and standard deviation (*SD*) or standard error (*SE*) when possible. The missing *SD* values were calculated from reported *SE* or coefficient of variation (*CV*) as shown in Equations 1 and 2 (Jian et al., 2020; Zhang et al., 2026):


(1) $$\mathrm{SD}=\mathrm{SE}\times \sqrt{n}$$

(2) SD=Mean×CV

**Figure 1:**
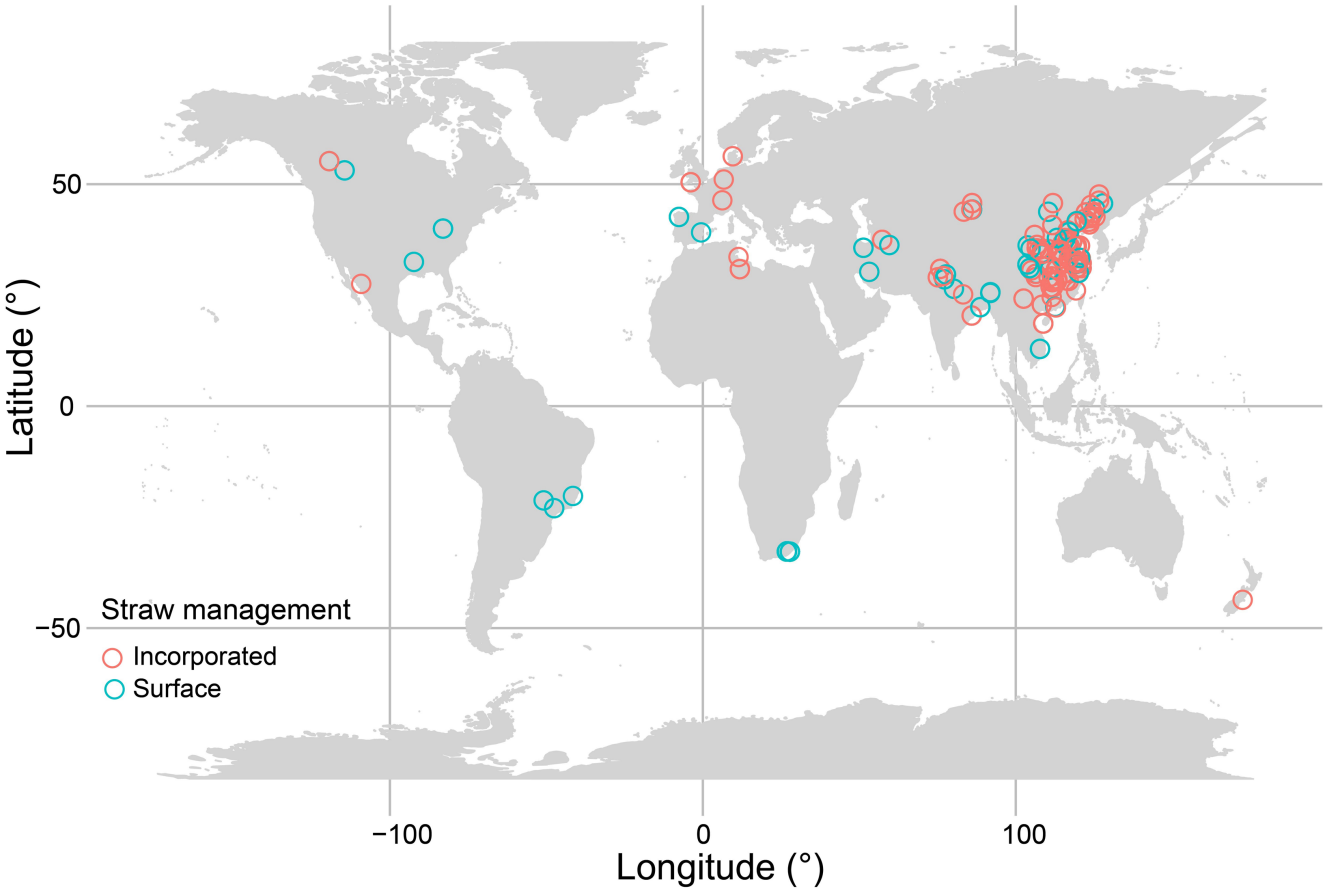
Global distribution of straw return included in this meta-analysis. Different colors denote straw management (i.e., incorporated and surface).

If data were only presented graphically, values were extracted using WebPlotDigitizer.^3^ When critical information was not provided in the article, we contacted the corresponding author to obtain this information. Figure 1 shows the geographical distribution of straw return experiments.

Our dataset comprised environmental and experimental variables, including latitude, longitude, elevation, mean annual precipitation (MAP), mean annual temperature (MAT), soil properties, straw characteristics, and field management information. Whenever MAP and MAT were not reported, these data were extracted from WorldClim 2.1.^4^ Google Earth^5^ was used to determine the unreported coordinates of experimental locations. For soil properties, the unreported initial soil parameters, such as, soil clay content, soil organic carbon, soil total nitrogen, and soil pH, were extracted using the website https://soilgrids.org/. Crop straw biochemical characteristics were extracted from the original articles. When these data were not reported in the article, mean values were collected from the straw quality dataset for the same species or group of species (Thiébeau et al., 2021). For field management, crop types were grouped into maize, rice, wheat, and other; straw management was grouped into incorporated and surface-applied; fertilizer form was grouped by urea and mixed; straw types were classified as green plant biomass, mature aboveground biomass, senescent plant biomass, and straw (Abalos et al., 2022). Moreover, straw rates were not considered in this study because of large variations in the amounts of straw used (Berhane et al., 2020).

### 2.2 Straw characteristics and categorical groups

Straw biochemical characteristics are cellulose, hemicellulose, neutral detergent soluble (soluble NDS fraction), water soluble carbon (WSC), and lignin (Zhang et al., 2026) in this meta-analysis. Cellulose, hemicellulose, and lignin relative contents, indicative of the composition of the insoluble residue fraction, were used; the difference between their sum and 100% was considered the soluble NDS fraction. The WSC determined after water extraction was expressed as a percentage of total carbon. The lignocellulose index (LCI) can be used as a criterion to show the recalcitrance of the plant cell wall (Herman et al., 2008). LCI was calculated following Li et al. (2025).

### 2.3 Soil C pool and Hy-EEAs

Soil C pool [i.e., soil microbial biomass C (MBC), soil organic C (SOC), soil dissolved organic C (DOC), soil easily oxidizable C (EOC), soil light fraction organic C (LFOC), and soil particulate organic C (POC)] and Hy-EEAs [*α*-1,4-glucosidase (AG, EC3.2.1.20), *β*-1,4-glucosidase (BG, EC3.2.1.21), *β*-1,4-xylosidase (BX, EC3.2.1.37), and β-d-cellobiosidase (CBH, EC3.2.1.91)] were included in this meta-analysis. Based on data availability criteria, HFOC was excluded from the meta-analysis as the number of comparable studies reporting this fraction was too limited to support meaningful statistical synthesis. If a study only reported soil organic matter (SOM) content, SOC was calculated as shown in Equation 3 (Gattinger et al., 2012):


(3) $$\mathrm{SOC}=\frac{\mathrm{SOM}}{1.72}$$

If a study reported more than one type of Hy-EEAs, their sum values were considered the overall responses of Hy-EEAs (see Supplementary Materials and Methods). If a study reported multiple straw return responses (i.e., more than one straw return treatment), each treatment was included separately in our dataset. We also recorded soil pH for both no-straw-return and straw return treatments when these data were reported. All original data used in this meta-analysis are available from the figshare (Li S. L. et al., 2022).

### 2.4 Meta-analysis, model selection, and regression analysis

A meta-analysis was used to evaluate the effects of straw return on Hy-EEAs and soil C pool, and other ancillary variables (Hedges et al., 1999). We calculated the logarithmic response ratio (*lnR*) and its variance for each observation to synthesize the effect of straw return on Hy-EEAs and soil C pool as shown in Equation 4:


(4) $$\ln R=\ln(\frac{X_{S}}{X_{C}})=\ln R(X_{S})-\ln(X_{C})$$

where *X_S_* and *X_C_* are the arithmetic mean values in the straw return and no-straw-return treatments, respectively.

The variances (*ν*) of *lnR* were calculated as shown in Equation 5 (Chen et al., 2018; Li et al., 2025):


(5) $$v=\frac{S_{S}^{2}}{n_{S}X_{S}^{2}}+\frac{S_{C}^{2}}{n_{C}X_{C}^{2}}$$

where $n_{S}$ and $n_{C}$ refer to the number of replicates and *S_S_* and *S_C_* are the *SD* for straw return and no-straw-return treatments, respectively.

We calculated effect sizes using the *escalc* function in *metafor* package (Viechtbauer, 2010). Overall effect size in a weighted mixed-effects model was calculated using *rma.mv* function from the *metafor* package (Viechtbauer, 2010). The overall effect size was transformed into percentage change, that is, (e*^lnR^* − 1) × 100%. The overall effect of straw return on each response variable was considered significant if the *p*-value was < 0.05.

A random-forest model selection in the context of meta-analysis was used to determine the most important predictors of the effect of straw return on the studied variables. We trained a random-forest meta-analysis with preselected predictors and calculated variable importance with *metaforest* (Van Lissa, 2017). Model selection analysis in the *glmulti* R package was used to determine the important predictors of the *lnR* of Hy-EEAs and soil C pool (Calcagno and de Mazancourt, 2010). Possible combinations of the environmental and experimental variables (e.g., latitude, MAT, MAP, soil pH, soil clay content, soil C:N ratios, straw management, and fertilizer form) were incorporated into the model selection analysis. Model selection was based on the Akaike Information Criterion. The relative importance of each variable for a certain model was estimated as the sum of Akaike weights of all predictors in this model. A threshold of 0.8 was used to differentiate the important and unimportant predictors.

## 3 Results

**Figure 2:**
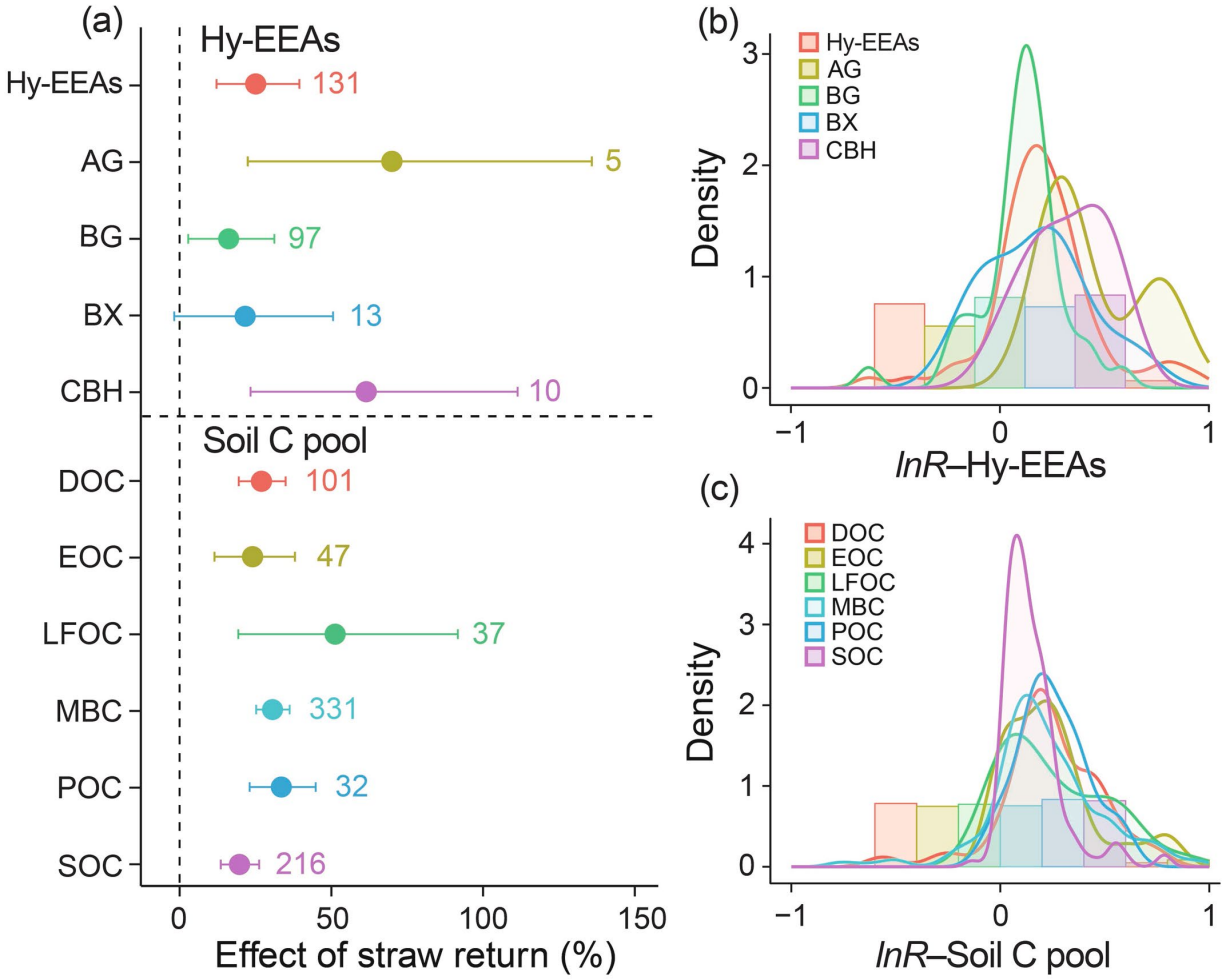
**(a)** Effect of straw return on Hy-EEAs and soil C pool. **(b)** Distribution of the log-transformed response ratios of Hy*-*EEAs (*lnR*–Hy*-*EEAs) to straw return. **(c)** Distribution of the log-transformed response ratios of soil carbon (C) pools (*lnR*–C pool) to straw return. Numbers refer to the sample size for each variable, and error bars indicate 95% confidence intervals. Hy-EEAs, hydrolytic carbon-degrading enzyme activities; AG, *α*-1,4-glucosidase; BG, *β*-1,4-glucosidase; BX, β-1,4-xylosidase; CBH, β-D-cellobiosidase. Soil C pool refers to soil dissolved organic C (DOC), easily oxidizable C (EOC), light fraction organic C (LFOC), microbial biomass C (MBC), particulate organic C (POC), and soil organic C (SOC).

Averaged across the whole dataset, straw return significantly enhanced Hy-EEAs and C content in soils (Figure 2). It is specific that straw return significantly increased AG activity by 70%, BG activity by 16%, and CBH activity by 62%, but they had no effect on BX activity (Figure 2a). The response of Hy-EEAs to straw return was normally distributed (Figure 2b). Regarding soil C pool, straw return significantly increased DOC by 27%, EOC by 24%, LFOC by 51%, MBC by 31%, POC by 34%, and SOC storage by 20% compared to no-straw-return treatment (Figure 2a). Moreover, the response of soil C pool to straw return was normally distributed (Figure 2c).

**Figure 3:**
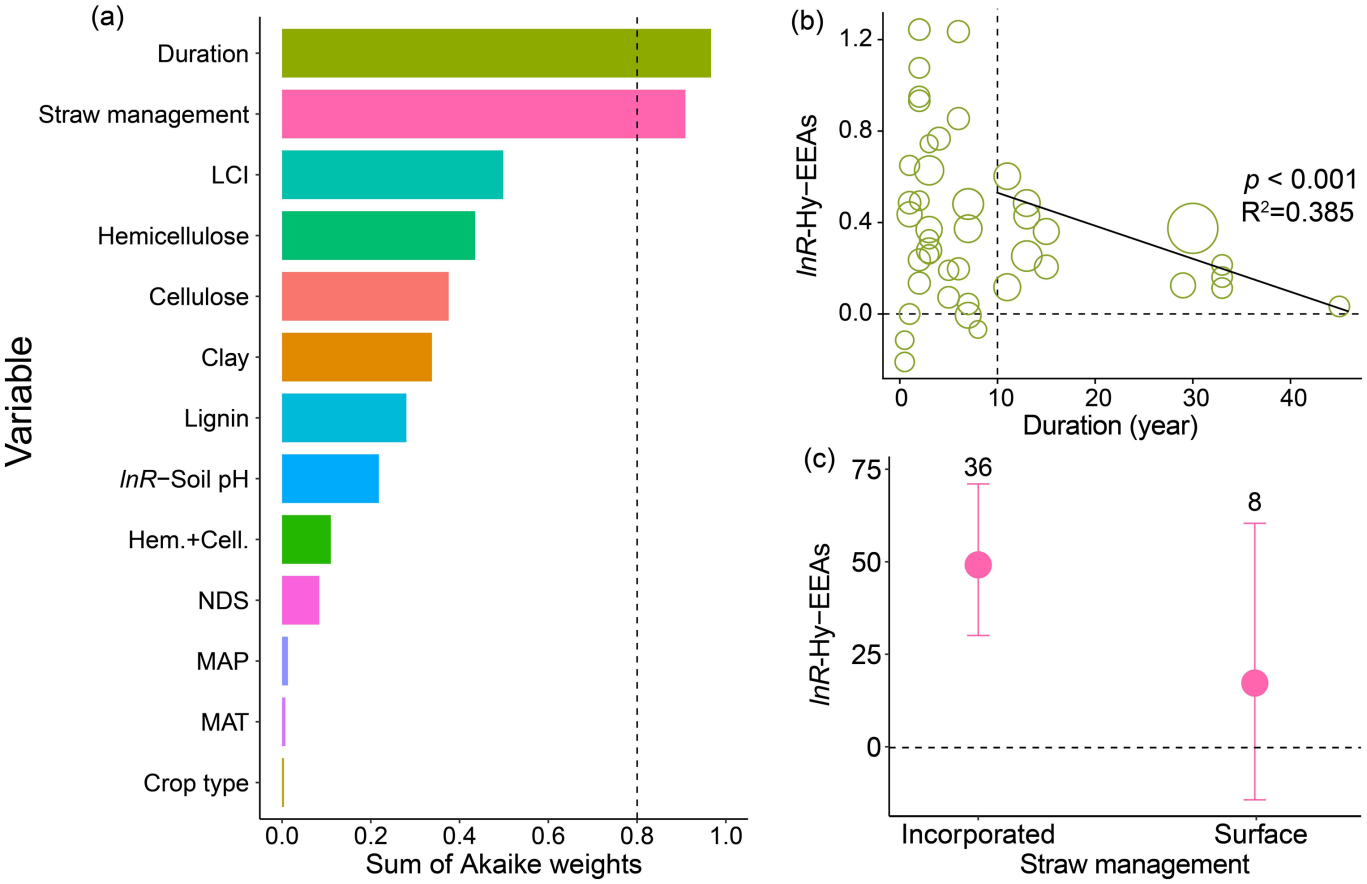
**(a)** Relative importance of predictors regulating the effect of straw return on soil Hy-EEAs. **(b)** Relationships between straw return-induced changes in soil Hy-EEAs and experiment duration. **(c)** The effects of straw return on soil Hy-EEAs grouped by different straw management. Error bars show 95% confidence intervals, and the numbers above the error bars indicate sample sizes. Hy-EEAs, hydrolytic C-degrading enzyme activities; LCI, lignocellulose index; straw management, incorporated and surface; clay, soil clay content (%); lignin, straw lignin content (% DM); NDS, soluble NDS; *lnR*-soil pH, straw return-induced changes in soil pH; Hem. + Cell., the sum of hemicellulos and cellulose; MAP, mean annual precipitation; MAT, mean annual temperature; crop type, maize, rice, wheat, and other.

Our random-meta-forest approach identified soil clay content, MAP, crop type, experiment duration, MAT, and straw management as the most important predictors of straw return effects on Hy-EEAs (Supplementary Figures S2, S3). Model selection analyses identified that experiment duration and straw management were the important predictors of straw return effect on Hy-EEAs (Figure 3a). Specifically, straw return effects on Hy-EEAs decreased with experiment duration greater than 10 years, while no relationship was found for duration less than 10 years (Figure 3b). Incorporated straw significantly increased soil Hy-EEAs by 14% (49%; 95% CIs: 30.1–70.1%), while no effect was observed for surface-applied straw (17%; 95% CIs: −14.3–60.4%) (Figure 3c). The amount of changes in response of Hy-EEAs to straw return was significantly positive for crop type, fertilizer form, and straw type (*p* < 0.001; Supplementary Figure S8).

There was a significant positive relationship between *lnR* of MBC content and *lnR* of Hy*-*EEAs (*R*^2^ = 0.18, *p* < 0.001). Our random-meta-forest approach identified soil clay content, *lnR* of Hy*-*EEAs, MAP, experiment duration, crop type, MAT, and straw management as the most important predictors of straw return effects on MBC content (Supplementary Figures S4, S5). Changes of MBC content in response to straw return were significantly negative when straw types were green plant biomass and senescent plant but significantly positive when straw types were mature aboveground biomass (*p* < 0.05; Supplementary Figure S9).

The response of Hy*-*EEAs to straw return was positively correlated with straw return-induced changes in SOC storage (*R*^2^ = 0.21; *p* < 0.05). Our random-meta-forest approach identified MAP, experiment duration, soil clay content, MAT, *lnR* of Hy*-*EEAs, and straw management as the most significant predictors of straw return effects on SOC storage (Supplementary Figures S6, S7). The response of Hy*-*EEAs also explained further changes in the response of soil C pool compared to a wide range of additional factors considered in the analysis (Table 1). Changes of Hy-EEAs in response to straw return were significantly positive for maize, rice, wheat, mixed fertilization, urea, straw, surface-applied straw, and incorporated straw (*p* < 0.05; Supplementary Figure S10).

**Table 1:** Evaluation of model parameters used to explain soil C pool (DOC, EOC, MBC, LFOC, SOC, and POC) under straw return.

Soil carbon pools	Variable	*F*	*R* ^2^	SE	*t*	*df*	*p*	*n*
DOC	*lnR*–pH	1.195	0.018	0.636	−1.093	18	0.289	20
	*lnR*–Hy-EEAs	0.077	0.016	0.158	−0.277	23	0.784	25
	*lnR*–AG	7.887	1.000	0.320	2.808	1	0.218	2
	*lnR*–BG	0.103	0.074	0.255	0.320	17	0.753	19
	***lnR*–BX**	**18.628**	**0.834**	**0.241**	**4.316**	**5**	**0.008**	**7**
	*lnR*–CBH	4.529	–	0.345	−2.128	1	0.280	3
EOC	***lnR*–pH**	**32.730**	**0.914**	**2.984**	**−5.721**	**7**	**0.001**	**9**
	*lnR*–Hy-EEAs	0.590	0.074	0.142	0.768	10	0.460	12
	*lnR*–AG	7.887	1.000	0.320	2.808	1	0.218	2
	***lnR*–BG**	**6.664**	**0.647**	**0.193**	**2.582**	**10**	**0.027**	**12**
	***lnR*–BX**	**22.777**	**0.007**	**0.106**	**−4.773**	**3**	**0.018**	**5**
	*lnR*–CBH	4.529	0.000	0.345	−2.128	1	0.280	3
MBC	*lnR*–pH	0.001	0.001	0.343	−0.029	76	0.977	78
	***lnR*–Hy-EEAs**	**8.818**	**0.174**	**0.076**	**2.970**	**80**	**0.004**	**62**
	*lnR*–AG	10.606	1.000	0.401	3.257	1	0.190	2
	*lnR*–BG	3.172	0.088	0.097	1.781	60	0.080	62
	*lnR*–BX	1.527	0.460	0.357	1.236	4	0.284	6
	*lnR*–CBH	2.856	0.503	0.302	1.690	3	0.190	5
LFOC	***lnR*–pH**	**26.579**	**0.734**	**4.964**	**−5.155**	**8**	**0.001**	**10**
	*lnR*–Hy-EEAs	0.081	0.022	0.234	−0.285	8	0.783	10
	*lnR*–AG	7.887	1.000	0.320	2.808	1	0.218	2
	*lnR*–BG	0.653	0.054	0.342	0.808	8	0.442	10
	*lnR*–BX	0.757	0.128	0.506	0.870	5	0.424	7
	*lnR*–CBH	4.529		0.345	−2.128	1	0.280	3
SOC	*lnR*–pH	0.607	0.001	0.261	0.779	66	0.439	68
	***lnR*–Hy-EEAs**	**9.606**	**0.098**	**0.104**	**3.099**	**40**	**0.004**	**42**
	*lnR*–AG	0.044	0.016	0.330	0.210	1	0.868	3
	***lnR*–BG**	**14.139**	**0.176**	**0.127**	**3.760**	**36**	**0.001**	**38**
	*lnR*–BX	0.894	0.263	0.467	0.946	2	0.444	4
	*lnR*–CBH	0.187	0.000	0.217	−0.432	4	0.688	6
POC	*lnR*–pH	1.479	0.010	4.219	−1.216	10	0.252	12
	*lnR*–Hy-EEAs	0.005	0.048	0.157	−0.068	7	0.948	9
	*lnR*–AG	3.771	1.000	0.424	1.942	1	0.303	2
	*lnR*–BG	2.669	0.388	0.158	1.634	7	0.146	9
	***lnR*–BX**	**12.792**	**0.499**	**0.181**	**3.577**	**5**	**0.016**	**7**
	*lnR*–CBH	2.300	0.860	0.487	−1.517	1	0.371	3

Model predictors with significant correlation (*p* < 0.05) showed in bold. df, denominator degree of freedom. DOC, soil dissolved organic C; EOC, easily oxidizable C; LFOC, light fraction organic C; MBC, microbial biomass C; POC, particulate organic C; SOC, soil organic C. lnR, log-transformed response ratio; Hy-EEAs, soil hydrolytic C-degrading extracellular enzyme activities; AG, *α*-1,4-Glucosidase (E.C.3.2.1.20); BG, *β*-1,4-Glucosidase (E.C.3.2.1.21); BX, *β*-1,4-Xylosidase (E.C.3.2.1.37); CBH, *β*-d-Cellobiosidase (E.C.3.2.1.91). Symbol “−” indicate that the model analysis was limited to studies that concurrently reported soil carbon and the Hy-EEAs. These parameters were reported for only subsets of studies and therefore were analyzed individually.

## 4 Discussion

### 4.1 Changes in Hy-EEAs with straw return

**Figure 4:**
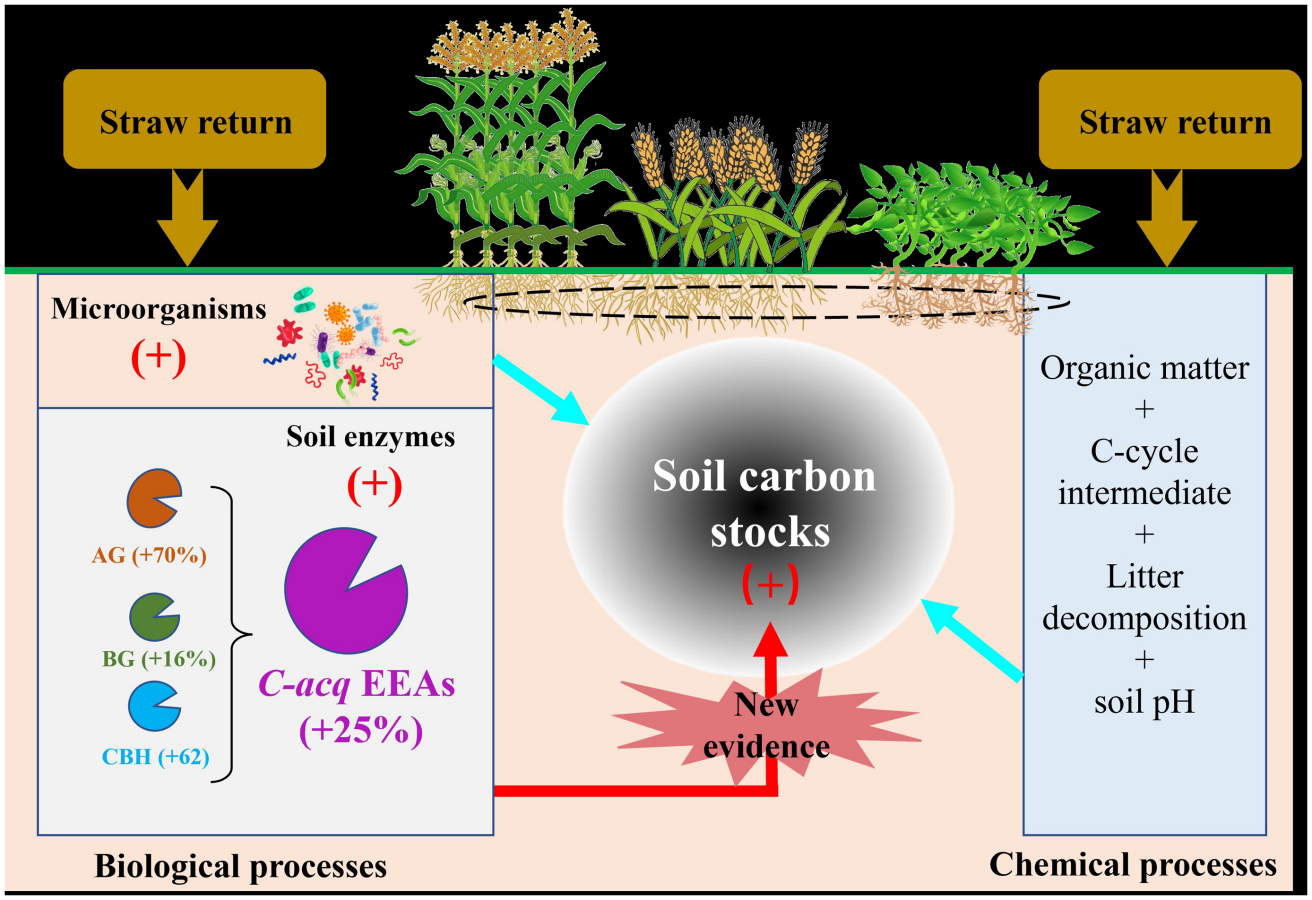
Schematic diagram illustrating the effects of straw return on soil Hy-EEAs and soil C pool and their relationships with soil carbon increase in agricultural systems. Hy-EEAs, hydrolytic carbon-degrading enzyme activities; AG, α-1,4-glucosidase; BG, β-1,4-glucosidase; CBH, β-D-cellobiosidase.

Straw return-induced increase in soil Hy-EEAs reveals that soil microorganisms help decompose additional C inputs by stimulating the production of extracellular enzymes (Sinsabaugh, 2010; Zhang et al., 2016). Indeed, several studies have reported that straw return favors soil microbial functional communities degrading the additional C inputs (Henriksen and Breland, 1999; Zhao X. M. et al., 2016). In this study, we propose four possible explanations (Figure 4).

First, shifts in C substrate availability due to straw return may be the explicit mechanism to explain this change. This is because soil Hy-EEAs could hydrolyze labile C substrates (Ma et al., 2019; Zuo et al., 2022). Root exudates and plant litter are important sources of hydrolyzable C (Zechmeister-Boltenstern et al., 2015). Straw return can increase crop growth and thus increase the production of root exudates and plant litter (Liu et al., 2014; Xia et al., 2018), which in turn increases the availability of labile C substrates. Therefore, to support microbial metabolic activities, soil microbes might increase soil Hy-EEAs to use existing pools of labile C substrates (Chen et al., 2020; Cotrufo et al., 2013).

Second, a shift in soil nutrient contents with straw return could help explain the changes in Hy-EEAs. Straw return can enhance soil nutrient contents, such as soil nitrogen and soil C, promoting crop growth (Xia et al., 2018). Straw return stimulation of crop growth would also change root community composition, forming more soil microorganisms and soil organic matter (Huang et al., 2021; Li et al., 2020; Wingeyer et al., 2012). Besides, straw return-stimulation of soil temperature and soil water would enhance the soil organic matter mineralization and soil nutrient availability (Jarvis et al., 1996; Stemmer et al., 1999). Increased soil nutrient availability with straw return could alleviate plant–microbial competition for nutrients and increase nutrient storage capacity to fuel the production of Hy-EEAs (Liu et al., 2014; Xia et al., 2018).

Third, soil clay content could account for shifts in Hy-EEAs (Supplementary Figures S2, S3). Soil texture usually reflects soil aeration, with soil clay content having an important role in soil water-holding capacity (Liu et al., 2014; Yu et al., 2018). Previous studies have shown that soil clay content is a key soil property affecting the soil’s capacity to store C (Jagadamma and Lal, 2010; Six et al., 2004). Importantly, straw return is more conducive to decreasing clay dispersion (Huang et al., 2021; Liu et al., 2014), which will prevent structurally complex macromolecules and decrease their accessibility to soil microbes, potentially increasing the production of Hy-EEAs (Fenner and Freeman, 2020).

Fourth, shifts in soil pH may be one of the predictors of the variation in the response of Hy-EEAs to straw return (Supplementary Figure S1). The predominant role of soil pH has been reported in published studies at local sites and/or large spatial regions (Bahram et al., 2018; Shu et al., 2022; Zheng et al., 2019). There are associations between soil pH and other soil characteristics (Högberg et al., 2007). For example, soil pH is a major factor influencing the structure of the soil microbial community (Fierer and Jackson, 2006; Nilsson et al., 2007; Powlson et al., 2011; Zak et al., 2003). Straw return affects soil pH (Islam et al., 2022), making the soil more suitable for microbial community composition and growth (Zak et al., 2003). Therefore, straw return-induced changes in enzyme and microbial activities could increase soil Hy-EEAs (Fierer and Jackson, 2006; Nilsson et al., 2007; Powlson et al., 2011).

Our results indicated that straw return significantly increased soil microbial biomass carbon (MBC) content (*p* < 0.05; Figure 2a). This increase can be explained by several interconnected mechanisms: (1) Straw provides a direct labile carbon source, stimulating microbial growth and activity. Subsequent production of microbial by-products promotes the formation and stabilization of soil macroaggregates, which can physically protect microbial biomass and reduce its turnover, leading to net MBC accumulation (Jastrow, 1996; Kalbitz et al., 2000). (2) By altering soil physical conditions, surface straw may limit oxygen diffusion and slow the decomposition rate. This suppression of aerobic mineralization can shift the microbial community toward groups with higher biomass efficiency or slower turnover, thereby increasing MBC (Zhu et al., 2015). (3) Straw incorporation rapidly modifies the pool sizes of labile soil organic carbon fractions. This shift in resource availability stimulates microbial growth and can result in a short-term elevation of MBC (Roper et al., 2010; Singh et al., 2007). (4) Straw amendment can enhance crop root growth and rhizodeposition. The increased input of readily available root-derived carbon further supports microbial growth in the rhizosphere, contributing to higher MBC (Li S. L. et al., 2022; Maarastawi et al., 2019).

In our study, straw-induced increases in Hy-EEAs were positively correlated with soil MBC content (Table 1). Why does the effect of straw return on MBC content increase with increasing Hy-EEAs? The degradation of labile C substrates requires less energy than the degradation of structurally complex macromolecule substrates (Mooshammer et al., 2017; Sinsabaugh, 2010). In addition, soil microbes can adjust their community composition or change their C utilization strategies to adapt to straw return (Huang et al., 2021; Jensen et al., 1997; Ocio et al., 1991; Xia et al., 2018). Alternatively, straw return could help restructure the microbial community and change litter quality, promoting the microbial production of Hy-EEAs (Liu et al., 2014; Su et al., 2020).

Our results show that straw return significantly enhanced SOC storage. Importantly, straw return-induced increases in Hy-EEAs were positively correlated with straw return responses of SOC storage. This is primarily due to the fact that straw return increased C allocation for microbial growth by enhancing soil enzyme activity, which would increase SOC storage (Diacono and Montemurro, 2011; Guo et al., 2018). The increase in C allocation for microbial growth will increase the microbial C residual available for soil C (Martens et al., 1992; Wang et al., 2021). Furthermore, straw return could increase the microbial C use efficiency, i.e., the ratio of C allocated for growth to C allocated for respiration (Allison et al., 2007; Manzoni et al., 2012; Rath and Rousk, 2015). Another explanation may be that straw return will likely decrease the accessibility of newly generated microbial C residue and formerly protected SOM by stimulating Hy-EEAs to degrade less chemically complex macromolecules (Chen et al., 2020; Luo et al., 2018; Mueller et al., 2020; Sinsabaugh and Shah, 2012). The decomposition of these uncomplicated macromolecules would release some associated physically and/or chemically related N and phosphorus (P), which may amplify the effect of straw return on SOM decomposition due to increased nutrient availability to microbial decomposers (Chen et al., 2018; Lavallee et al., 2020).

The positive correlation between straw return effects on SOC storage and Hy-EEAs (Table 1) indicates that straw return effects on SOC storage can be explained by soil enzyme responses. Indeed, soil C stocks are affected by the balance between crop litter that is decomposed and transformed into soil organic matter versus the amount that is mineralized (Cotrufo et al., 2015; Kallenbach et al., 2016). Thus, other soil processes, for example, straw return-induced shifts in root exudation, litter input, the formation of stable soil organic matter from microbial products, and C leaching, would also contribute to shifts in SOC storage with straw return (Liang et al., 2019; Luo et al., 2017; Pausch and Kuzyakov, 2018), while those potential processes are not evaluated in this meta-analysis. Besides, even though the enzymes analyzed in this meta-analysis could indirectly affect soil C decomposition in bulk soil, they are involved in the decomposition of particulate soil organic matter and plant litter (Lavallee et al., 2020; Kuzyakov, 2010; Soong et al., 2020; Zechmeister-Boltenstern et al., 2015). Therefore, although straw return-induced changes in Hy-EEAs play a critical role in determining soil C dynamics with straw return, the microbial mechanisms underlying shifts in enzyme activities remain unclear.

### 4.2 Implications and uncertainties

Understanding the effects of straw return on C in soils and Hy-EEAs and revealing the key mechanisms between them will help improve predictions of below-ground C dynamics of the future in agricultural systems. Our meta-analysis provided important information for the test and development of biogeochemistry models, further predicting the potential mechanisms for soil C cycling and turnover. Several critical uncertainties with respect to further studies still exist. First, since the differences in straw amount and straw type differ among cropping systems and vary substantially across different experiment sites (Berhane et al., 2020), this heterogeneity might pose a major challenge to the prediction of microbial EEAs, microbial processes, and soil C cycling under straw return.

Second, few studies simultaneously measured soil enzyme activities and soil C content under straw return in the same experimental platform. Therefore, to advance our understanding of the relationships between soil enzyme activities and C in soils under straw return, we strongly encourage agricultural researchers to observe enzyme and soil C stocks concurrently.

Third, the microbial mechanisms’ potential shifts in soil enzyme activities remain unclear, hampering the incorporation of microbial mechanisms in the models (Chen et al., 2019). Although incorporating microbial processes can promote the performance of Earth System Models (Allison et al., 2010; Wieder et al., 2013), the representation of microbial processes in these models varies and is disputed (Treseder et al., 2012). Therefore, we suggest that future research quantifies the associations between gene expression, microbial community composition, and soil enzyme activities.

Fourth, straw return might interactively and simultaneously change the soil characteristics (e.g., soil pH, soil moisture, and microbial biomass) (Liu et al., 2014; Xia et al., 2018), which could potentially modify Hy-EEAs and further mediate soil C cycling. These changes may render the impacts of straw return on soil C dynamics and Hy-EEAs are highly complex but deserve to be further explored and studied.

## 5 Conclusion

In the current synthesis, straw return significantly enhanced the activities of AG, BG, and CBH but had no effect on BX activity. While incorporated straw significantly increased soil phosphatase activity by 28%, long-term N loading had no significant effect. Straw return effects on Hy-EEAs increased with incorporated straw but not with surface-applied straw. Moreover, straw return significantly increased DOC, EOC, LFOC, MBC, POC, and SOC storage, suggesting that the inclusion of straw return into agricultural systems can potentially increase soil C sequestration. The regression analyses indicated that the responses of MBC content and SOC storage were positively correlated with straw return stimulation of Hy-EEAs. However, there were no clear relationships between the response ratios (*lnR*) of DOC, EOC, LFOC, and POC and *lnR* of Hy-EEAs. These different trends were affected by environmental conditions (MAT and MAP) and soil properties (e.g., soil pH). This study may help understand the effects of straw return on C in soils and Hy-EEAs, providing novel insights into the potential relationships between below-ground C dynamics and Hy-EEAs.

## Data Availability

The datasets presented in this study can be found in online repositories. The names of the repository/repositories and accession number(s) can be found in the article/Supplementary material.

## Footnotes

1
http://apps.webofknowledge.com/

2
http://www.cnki.net/

3
https://automeris.io/WebPlotDigitizer/

4
https://www.worldclim.org/

5
https://earth.google.com

## References

[ref1] Abalos D. Rittl T. F. Recous S. Thiébeau P. Topp C. F. E. van Groenigen K. J. . (2022). Predicting field N2O emissions from crop residues based on their biochemical composition: a meta-analytical approach. Sci. Total Environ. 812:152352. doi: 10.1016/j.scitotenv.2021.152532 34952057

[ref2] Allison S.D. Gartner T.B. Holland K. Weintraub M. Sinsabaugh R.L. 2007 Soil enzymes: linking proteomics and ecological processes. Manual Environ. Microbiol. 704–711. doi: 10.1128/9781555815882.ch58

[ref3] Allison S. D. Wallenstein M. D. Bradford M. A. (2010). Soil-carbon response to warming dependent on microbial physiology. Nat. Geosci. 3, 336–340. doi: 10.1038/ngeo846

[ref4] Bahram M. Hildebrand F. Forslund S. K. Anderson J. L. Soudzilovskaia N. A. Bodegom P. M. . (2018). Structure and function of the global topsoil microbiome. Nature 560, 233–237. doi: 10.1038/s41586-018-0386-6, 30069051

[ref5] Bardgett R. D. Freeman C. Ostle N. J. (2008). Microbial contributions to climate change through carbon cycle feedbacks. ISME J. 2, 805–814. doi: 10.1038/ismej.2008.58, 18615117

[ref6] Berhane M. Xu M. Liang Z. Shi J. Wei G. Tian X. (2020). Effects of long-term straw return on soil organic carbon storage and sequestration rate in North China upland crops: a meta-analysis. Glob. Change Biol. 26, 2686–2701. doi: 10.1111/gcb.15018, 31991046

[ref7] Bhattacharyya P. Roy K. S. Neogi S. Chakravorti S. P. Behera K. S. Das K. M. . (2012). Effect of long-term application of organic amendment on C storage in relation to global warming potential and biological activities in tropical flooded soil planted to rice. Nutr. Cycl. Agroecosyst. 94, 273–285. doi: 10.1007/s10705-012-9540-y

[ref8] Bray S. R. Kitajima K. Mack M. C. (2012). Temporal dynamics of microbial communities on decomposing leaf litter of 10 plant species in relation to decomposition rate. Soil Biol. Biochem. 49, 30–37. doi: 10.1016/j.soilbio.2012.02.009

[ref9] Burns R. G. DeForest J. L. Marxsen J. Sinsabaugh R. L. Stromberger M. E. Wallenstein M. D. . (2013). Soil enzymes in a changing environment: current knowledge and future directions. Soil Biol. Biochem. 58, 216–234. doi: 10.1016/j.soilbio.2012.11.009

[ref10] Calcagno V. de Mazancourt C. (2010). Glmulti: an R package for easy automated model selection with (generalized) linear models. J. Stat. Softw. 34, 1–29. doi: 10.18637/jss.v034.i12

[ref11] Chen Y. Chen J. Luo Y. (2019). Data-driven ENZYme (DENZY) model represents soil organic carbon dynamics in forests impacted by nitrogen deposition. Soil Biol. Biochem. 138:107575. doi: 10.1016/j.soilbio.2019.107575

[ref12] Chen J. Elsgaard L. van Groenigen K. J. Olesen J. E. Liang Z. Jiang Y. . (2020). Soil carbon loss with warming: new evidence from carbon-degrading enzymes. Glob. Change Biol. 26, 1944–1952. doi: 10.1111/gcb.14986 31909849

[ref13] Chen J. Luo Y. García-Palacios P. Cao J. Dacal M. Zhou X. . (2018). Differential responses of carbon-degrading enzyme activities to warming: implications for soil respiration. Glob. Chang. Biol. 24, 4816–4826. doi: 10.1111/gcb.14394, 29999577

[ref14] Cherubin M. R. de Silva Oliveira D. M. Feigl B. J. Pimentel L. G. Lisboa I. P. Gmach M. R. . (2018). Crop residue harvest for bioenergy production and its implications on soil functioning and plant growth: a review. Sci. Agric. 75, 255–272. doi: 10.1590/1678-992x-2016-0459

[ref15] Cotrufo M. F. Soong J. L. Horton A. J. Campbell E. E. Haddix M. L. Wall D. H. . (2015). Formation of soil organic matter via biochemical and physical pathways of litter mass loss. Nat. Geosci. 8, 776–779. doi: 10.1038/ngeo2520

[ref16] Cotrufo M. F. Wallenstein M. D. Boot C. M. Denef K. Paul E. (2013). The microbial efficiency-matrix stabilization (MEMS) frame-work integrates plant litter decomposition with soil organic matter stabilization: do labile plant inputs form stable soil organic matter? Glob. Chang. Biol. 19, 988–995. doi: 10.1111/gcb.12113C, 23504877

[ref17] Dai W. Fang K. Gao H. Wang J. Penttinen P. Sha Z. . (2022). Differential responses of soil organic carbon fractions and carbon turnover related enzyme activities to wheat straw incorporation in subtropical China. Phyton 91:169. doi: 10.32604/phyton.2022.016407

[ref18] Davidson E. A. Janssens I. A. (2006). Temperature sensitivity of soil carbon decomposition and feedbacks to climate change. Nature 440, 165–173. doi: 10.1038/nature04514, 16525463

[ref19] Deng S. P. Tabatabai M. A. (1995). Cellulase activity of soils: effect of trace elements. Soil Biol. Biochem. 27, 977–979. doi: 10.1016/0038-0717(95)00005-Y

[ref20] Diacono M. Montemurro F. (2011). Long-term effects of organic amendments on soil fertility. Agron. Sustain. Dev. 30, 761–786. doi: 10.1007/978-94-007-0394-0_34

[ref21] Fenner N. Freeman C. (2020). Woody litter protects peat carbon stocks during drought. Nat. Clim. Chang. 10, 363–369. doi: 10.1038/s41558-020-0727-y

[ref22] Fierer N. Jackson R. B. (2006). The diversity and biogeography of soil bacterial communities. Proc. Natl. Acad. Sci. USA 103, 626–631. doi: 10.1073/pnas.0507535103, 16407148 PMC1334650

[ref23] Gattinger A. Muller A. Haeni M. Skinner C. Fliessbach A. Buchmann N. . (2012). Enhanced top soil carbon stocks under organic farming. Proc. Natl. Acad. Sci. 109, 18226–18231. doi: 10.1073/pnas.1209429109, 23071312 PMC3497757

[ref24] Guo J. Liu W. Zhu C. Luo G. Kong Y. Ling N. . (2018). Bacterial rather than fungal community composition is associated with microbial activities and nutrient-use efficiencies in a paddy soil with short-term organic amendments. Plant Soil 424, 335–349. doi: 10.1007/s11104-017-3547-8

[ref25] Hazarika S. Parkinson R. Bol R. Dixon L. Russell P. Donovan S. . (2009). Effect of tillage system and straw management on organic matter dynamics. Agron. Sustain. Dev. 29, 525–533. doi: 10.1051/agro/2009024

[ref26] Hedges L. V. Gurevitch J. Curtis P. S. (1999). The meta-analysis of response ratios in experimental ecology. Ecology 80, 1150–1156. doi: 10.1890/0012-9658(1999)080[1150:TMAORR]2.0.CO;2

[ref27] Henriksen T. M. Breland T. A. (1999). Nitrogen availability effects on carbon mineralization, fungal and bacterial growth, and enzyme activities during decomposition of wheat straw in soil. Soil Biol. Biochem. 31, 1121–1134. doi: 10.1016/S0038-0717(99)00030-9

[ref28] Herman J. Moorhead D. Berg B. (2008). The relationship between rates of lignin and cellulose decay in aboveground forest litter. Soil Biol. Biochem. 40, 2620–2626. doi: 10.1016/j.soilbio.2008.07.003

[ref29] Högberg M. N. Högberg P. Myrold D. D. (2007). Is microbial community composition in boreal forest soils determined by pH, C-to-N ratio, the trees, or all three? Oecologia 150, 590–601. doi: 10.1007/s00442-006-0562-5, 17033802

[ref30] Huang T. Yang N. Lu C. Qin X. Siddique K. H. M. (2021). Soil organic carbon, total nitrogen, available nutrients, and yield under different straw returning methods. Soil Tillage Res. 214:105171. doi: 10.1016/j.still.2021.105171

[ref31] Islam M. U. Guo Z. Jiang F. Peng X. (2022). Does straw return increase crop yield in the wheat-maize cropping system in China? A meta-analysis. Field Crop Res. 279:108447. doi: 10.1016/j.fcr.2022.108447

[ref32] Jagadamma S. Lal R. (2010). Distribution of organic carbon in physical fractions of soils as affected by agricultural management. Biol. Fertil. Soils 46, 543–554. doi: 10.1007/s00374-010-0459-7

[ref33] Jarvis S. C. Stockdale E. A. Shepherd M. A. Powlson D. S. (1996). Nitrogen mineralization in temperate agricultural soils: processes and measurement. Adv. Agron. 57, 187–235. doi: 10.1016/S0065-2113(08)60925-6

[ref34] Jastrow J. D. (1996). Soil aggregate formation and the accrual of particulate and mineral-associated organic matter. Soil Biol. Biochem. 28, 665–676. doi: 10.1016/0038-0717(95)00159-X

[ref35] Jensen L. S. Mueller T. Magid J. Nielsen N. E. (1997). Temporal variation of C and N mineralization, microbial biomass and extractable organic pools in soil after oilseed rape straw incorporation in the field. Soil Biol. Biochem. 29, 1043–1055. doi: 10.1016/S0038-0717(97)00014-X

[ref36] Jian J. S. Du X. Reiter M. S. Stewart R. D. (2020). A meta-analysis of global cropland soil carbon changes due to cover cropping. Soil Biol. Biochem. 143:107735. doi: 10.1016/j.soilbio.2020.107735

[ref37] Jian S. Y. Li J. Chen J. Wang G. Mayes M. A. Dzantor K. E. . (2016). Soil extracellular enzyme activities, soil carbon and nitrogen storage under nitrogen fertilization: a meta-analysis. Soil Biol. Biochem. 101, 32–43. doi: 10.1016/j.soilbio.2016.07.003

[ref9400] Jiang Q. W. Li Y. Yang Q. L. Yang L. Siddique K. H. M. (2026). Response of reactive nitrogen loss to straw return: a meta-analysis. J. Soils Sediments. 26:7. doi: 10.1007/s11368-025-04215-3

[ref38] Kalbitz K. Solinger S. Park J. H. Michalzik B. Matzner E. (2000). Controls on the dynamics of dissolved organic matter in soils: a review. Soil Sci. 165, 277–304. doi: 10.1097/00010694-200004000-00001

[ref39] Kallenbach C. M. Frey S. D. Grandy A. S. (2016). Direct evidence for microbial-derived soil organic matter formation and its ecophysiological controls. Nat. Commun. 7:13630. doi: 10.1038/ncomms13630, 27892466 PMC5133697

[ref40] Kuzyakov Y. (2010). Priming effects: interactions between living and dead organic matter. Soil Biol. Biochem. 42, 1363–1371. doi: 10.1016/j.soilbio.2010.04.003

[ref41] Lal R. (2004). Soil carbon sequestration impacts on global climate change and food security. Science 304, 1623–1627. doi: 10.1126/science.1097396 15192216

[ref42] Lal R. (2005). World crop residues production and implications of its use as a biofuel. Environ. Int. 31, 575–584. doi: 10.1016/j.envint.2004.09.005, 15788197

[ref43] Lavallee J. M. Soong J. L. Cotrufo M. F. (2020). Conceptualizing soil organic matter into particulate and mineral-associated forms to address global change in the 21st century. Glob. Change Biol. 26, 261–273. doi: 10.1111/gcb.14859, 31587451

[ref44] Li S. Chen J. Shi J. Tian X. Li X. Li Y. . (2017). Impact of straw return on soil carbon indices, enzyme activity, and grain production. Soil Sci. Soc. Am. J. 81, 1475–1485. doi: 10.2136/sssaj2016.11.0368

[ref45] Li S. L. Cui Y. X. Xia Z. Q. Zhang X. H. Zhu M. M. Gao Y. . (2022). The mechanism of the dose effect of straw on soil respiration: evidence from enzymatic stoichiometry and functional genes. Soil Biol. Biochem. 168:108636. doi: 10.1016/j.soilbio.2022.108636

[ref46] Li Y. Feng H. Chen J. Lu J. S. Wu W. J. Liu X. Z. . (2022). Biochar incorporation increases winter wheat (*Triticum aestivum* L.) production with significantly improving soil enzyme activities at jointing stage. Catena 211:105979. doi: 10.1016/j.catena.2021.105979

[ref47] Li Y. Lu J. Feng H. Chen J. Yang Q. (2025). Soil cellulase activity responds to straw return and correlates with soil organic carbon dynamics: a global meta-analysis. Plant Soil. doi: 10.1007/s11104-025-08223-7

[ref48] Li Y. Song D. Dang P. Wei L. Qin X. Siddique K. H. M. (2020). Combined ditch buried straw return technology in a ridge–furrow plastic film mulch system: implications for crop yield and soil organic matter dynamics. Soil Tillage Res. 199:104596. doi: 10.1016/j.still.2020.104596

[ref49] Li T. Sun Z. He C. Ge X. Ouyang Z. Wu L. (2020). Changes in soil bacterial community structure and microbial function caused by straw retention in the North China plain. Arch. Agron. Soil Sci. 66, 46–57. doi: 10.1080/03650340.2019.1593382

[ref50] Liang C. Amelung W. Lehmann J. Kästner M. (2019). Quantitative assessment of microbial necromass contribution to soil organic matter. Glob. Chang. Biol. 25, 3578–3590. doi: 10.1111/gcb.14781, 31365780

[ref51] Liang G. P. Wu H. J. Houssou A. A. Cai D. X. Wu X. P. Gao L. L. . (2018). Soil respiration, glomalin content, and enzymatic activity response to straw application in a wheat-maize rotation system. J. Soils Sediments 18, 697–707. doi: 10.1007/s11368-017-1817-y

[ref52] Liu C. Lu M. Cui J. Li B. Fang C. (2014). Effects of straw carbon input on carbon dynamics in agricultural soils: a meta-analysis. Glob. Chang. Biol. 20, 1366–1381. doi: 10.1111/gcb.12517, 24395454

[ref53] Lu F. E. Wang X. Han B. Ouyang Z. Duan X. Zheng H. U. . (2009). Soil carbon sequestrations by nitrogen fertilizer application, straw return and no-tillage in China’s cropland. Glob. Change Biol. 15, 281–305. doi: 10.1111/j.1365-2486.2008.01743.x

[ref54] Luo G. Li L. Friman V. P. Guo J. Guo S. Shen Q. . (2018). Organic amendments increase crop yields by improving microbe-mediated soil functioning of agroecosystems: a meta-analysis. Soil Biol. Biochem. 124, 105–115. doi: 10.1016/j.soilbio.2018.06.002

[ref55] Luo L. Meng H. Gu J. D. (2017). Microbial extracellular enzymes in biogeochemical cycling of ecosystems. J. Environ. Manag. 197, 539–549. doi: 10.1016/j.jenvman.2017.04.02, 28419976

[ref56] Ma Y. Li Liu D. Schwenke G. Yang B. (2019). The global warming potential of straw-return can be reduced by application of straw-decomposing microbial inoculants and biochar in rice-wheat production systems. Environ. Pollut. 252, 835–845. doi: 10.1016/j.envpol.2019.06.006, 31202136

[ref57] Maarastawi S. A. Frindte K. Bodelier P. L. Knief C. (2019). Rice straw serves as additional carbon source for rhizosphere microorganisms and reduces root exudate consumption. Soil Biol. Biochem. 135, 235–238. doi: 10.1016/j.soilbio.2019.05.007

[ref59] Majumder B. Mandal B. Bandyopadhyay P. K. Gangopadhyay A. Mani P. K. Kundu A. L. . (2008). Organic amendments influence soil organic carbon pools and rice-wheat productivity. Soil Sci. Soc. Am. J. 72, 775–785. doi: 10.2136/sssaj2006.0378

[ref60] Manzoni S. Taylor P. Richter A. Porporato A. Agren G. I. (2012). Environmental and stoichiometric controls on microbial carbon-use efficiency in soils. New Phytol. 196, 79–91. doi: 10.1111/j.1469-8137.2012.04225.x, 22924405

[ref61] Martens D. A. Johanson J. B. Frankenberger W. T. Jr. (1992). Production and persistence of soil enzymes with repeated addition of organic residues. Soil Sci. 153, 53–61. doi: 10.1097/00010694-199201000-00008

[ref62] Moher D. Liberati A. Tetzlaff J. Altman D. G. PRISMA Group (2009). Preferred reporting items for systematic reviews and meta-analyses: the PRISMA statement. Ann. Intern. Med. 151, 264–269. doi: 10.7326/0003-4819-151-4-200908180-00135, 19622511

[ref63] Mooshammer M. Hofhansl F. Frank A. H. Wanek W. Hämmerle I. Leitner S. . (2017). Decoupling of microbial carbon, nitrogen, and phosphorus cycling in response to extreme temperature events. Sci. Adv. 3:e1602781. doi: 10.1126/sciadv.1602781, 28508070 PMC5415334

[ref64] Mueller P. Granse D. Nolte S. Weingartner M. Hoth S. Jensen K. (2020). Unrecognized controls on microbial functioning in blue carbon ecosystems: the role of mineral enzyme stabilization and allochthonous substrate supply. Ecol. Evol. 10, 998–1011. doi: 10.1002/ece3.5962, 32015860 PMC6988540

[ref65] Nilsson L. O. Baath E. Falkengren-Grerup U. Wallander H. (2007). Growth of ectomycorrhizal mycelia and composition of soil microbial communities in oak forest soils along a nitrogen deposition gradient. Oecologia 153, 375–384. doi: 10.1007/s00442-007-0735-x, 17453252

[ref66] Ocio J. A. Brookes P. C. Jenkinson D. S. (1991). Field incorporation of straw and its effects on soil microbial biomass and soil inorganic N. Soil Biol. Biochem. 23, 171–176. doi: 10.1016/0038-0717(91)90131-3

[ref67] Pausch J. Kuzyakov Y. (2018). Carbon input by roots into the soil: quantification of rhizodeposition from root to ecosystem scale. Glob. Chang. Biol. 24, 1–12. doi: 10.1111/gcb.13850, 28752603

[ref68] Powlson D. S. Glendining M. J. Coleman K. Whitmore A. P. (2011). Implications for soil properties of removing cereal straw: results from long-term studies. Agron. J. 103, 279–287. doi: 10.2134/agronj2010.0146s

[ref69] Powlson D. S. Riche A. B. Coleman K. Glendining M. J. Whitmore A. P. (2008). Carbon sequestration in European soils through straw incorporation: limitations and alternatives. Waste Manag. 28, 741–746. doi: 10.1016/j.wasman.2007.09.024, 18061434

[ref70] Rath K. M. Rousk J. (2015). Salt effects on the soil microbial decomposer community and their role in organic carbon cycling: a review. Soil Biol. Biochem. 81, 108–123. doi: 10.1016/j.soilbio.2014.11.001

[ref71] Roper M. M. Gupta V. V. S. R. Murphy D. V. (2010). Tillage practices altered labile soil organic carbon and microbial function without affecting crop yields. Soil Res. 48, 274–285. doi: 10.1071/SR09143

[ref72] Shang Q. Yang X. Gao C. Wu P. Liu J. Xu Y. . (2011). Net annual global warming potential and greenhouse gas intensity in Chinese double rice-cropping systems: a 3-year field measurement in long-term fertilizer experiments. Glob. Change Biol. 17, 2196–2210. doi: 10.1111/j.1365-2486.2010.02374.x

[ref73] Sharma S. Vashisht M. Singh Y. Thind H. S. (2019). Soil carbon pools and enzyme activities in aggregate size fractions after seven years of conservation agriculture in a rice–wheat system. Crop Pasture Sci. 70, 473–485. doi: 10.1071/CP19013

[ref74] Shu X. Y. He J. Zhou Z. H. Xia L. L. Hu Y. F. Zhang Y. L. . (2022). Organic amendments enhance soil microbial diversity, microbial functionality and crop yields: a meta-analysis. Sci. Total Environ. 829:154627. doi: 10.1016/j.scitotenv.2022.154627 35306065

[ref75] Singh G. Jalota S. K. Singh Y. (2007). Manuring and residue management effects on physical properties of a soil under the rice-wheat system in Punjab, India. Soil Tillage Res. 94, 229–238. doi: 10.1016/j.still.2006.07.020

[ref76] Singh B. Shan Y. H. Johnson-Beebout S. E. Singh Y. Buresh R. J. (2008). Crop residue management for lowland rice-based cropping systems in Asia. Adv. Agron. 98, 117–199. doi: 10.1016/S0065-2113(08)00203-4

[ref77] Sinsabaugh R. L. (2010). Phenol oxidase, peroxidase and organic matter dynamics of soil. Soil Biol. Biochem. 42, 391–404. doi: 10.1016/j.soilbio.2009.10.014

[ref78] Sinsabaugh R. L. Findlay S. (1995). Microbial production, enzyme activity, and carbon turnover in surface sediments of the Hudson River estuary. Microb. Ecol. 30, 127–141. doi: 10.1007/BF00172569, 24185480

[ref79] Sinsabaugh R. L. Lauber C. L. Weintraub M. N. Ahmed B. Allison S. D. Crenshaw C. . (2008). Stoichiometry of soil enzyme activity at global scale. Ecol. Lett. 11, 1252–1264. doi: 10.1111/j.1461-0248.2008.01245.x, 18823393

[ref80] Sinsabaugh R. L. Shah J. J. F. (2012). Ecoenzymatic stoichiometry and ecological theory. Annu. Rev. Ecol. Evol. Syst. 43:313. doi: 10.1146/annurev-ecolsys-071112-124414

[ref81] Six J. Ogle S. M. Jay Breidt F. Conant R. T. Mosier A. R. Paustian K. (2004). The potential to mitigate global warming with no-tillage management is only realized when practised in the long term. Glob. Change Biol. 10, 155–160. doi: 10.1111/j.1529-8817.2003.00730.x

[ref82] Soong J. L. Fuchslueger L. Marañon-Jimenez S. Torn M. S. Janssens I. A. Penuelas J. . (2020). Microbial carbon limitation: the need for integrating microorganisms into our understanding of ecosystem carbon cycling. Glob. Chang. Biol. 26, 1953–1961. doi: 10.1111/gcb.14962, 31838767

[ref83] Stemmer M. Von Lützow M. Kandeler E. Pichlmayer F. Gerzabek M. H. (1999). The effect of maize straw placement on mineralization of C and N in soil particle size fractions. Eur. J. Soil Sci. 50, 73–85. doi: 10.1046/j.1365-2389.1999.00204.x

[ref84] Stewart C. E. Paustian K. Conant R. T. Plante A. F. Six J. (2007). Soil carbon saturation: concept, evidence and evaluation. Biogeochemistry 86, 19–31. doi: 10.1007/s10533-007-9140-0

[ref85] Su Y. Lv J. L. Yu M. Ma Z. H. Xi H. Kou C. L. . (2020). Long-term decomposed straw return positively affects the soil microbial community. J. Appl. Microbiol. 128, 138–150. doi: 10.1111/jam.14435, 31495045

[ref86] Sun J. Liang J. X. Kong D. J. Guo X. N. Wei Y. D. Zhou T. (2021). Effects of biochar and straw on the C:N:P stoichiometry of soil, microbes, and extracellular enzymes in an aeolian sandy soil. Acta Pratacult. Sin. 30:29. doi: 10.11686/cyxb2020407

[ref87] Tarnocai C. Canadell J. G. Schuur E. A. G. Kuhry P. Mazhitova G. Zimov S. (2009). Soil organic carbon pools in the northern circumpolar permafrost region. Glob. Biogeochem. Cycles 23. doi: 10.1029/2008GB003327

[ref88] Terrer C. Vicca S. Hungate B. A. Phillips R. P. Prentice I. C. (2016). Mycorrhizal association as a primary control of the CO2 fertilization effect. Science 353, 72–74. doi: 10.1126/science.aaf4610, 27365447

[ref89] Thiébeau P. Jensen L. S. Ferchaud F. Recous S. (2021). Dataset of biomass and chemical quality of crop residues from european areas. Data Brief 37:107227. doi: 10.1016/j.dib.2021.107227, 34189212 PMC8220339

[ref90] Tiemann L. Billings S. A. (2011). Indirect effects of nitrogen amendments on organic substrate quality increase enzymatic activity driving decomposition in a Mesic grassland. Ecosystems 14, 234–247. doi: 10.1007/s10021-010-9406-6

[ref91] Treseder K. K. Balser T. C. Bradford M. A. Brodie E. L. Dubinsky E. A. Eviner V. T. . (2012). Integrating microbial ecology into ecosystem models: challenges and priorities. Biogeochemistry 109, 7–18. doi: 10.1007/s10533-011-9636-5

[ref92] Trumbore S. E. Chadwick O. A. Amundson R. (1996). Rapid exchange between soil carbon and atmospheric carbon dioxide driven by temperature change. Science 272, 393–396. doi: 10.1126/science.272.5260.393

[ref93] Van Lissa C.J., 2017. MetaForest: exploring heterogeneity in meta-analysis using random forests. doi: 10.31234/osf.io/myg6s

[ref95] Viechtbauer W. (2010). Conducting meta-analyses in R with the metafor package. J. Stat. Softw. 36, 1–48. doi: 10.18637/jss.v036.i03

[ref96] Wallenstein M. D. Burns R. G. (2011). Ecology of extracellular enzyme activities and organic matter degradation in soil: a complex community-driven process. Methods Soil Enzymol. 9, 35–55. doi: 10.2136/sssabookser9.c2

[ref97] Wang B. An S. Liang C. Liu Y. Kuzyakov Y. (2021). Microbial necromass as the source of soil organic carbon in global ecosystems. Soil Biol. Biochem. 162:108422. doi: 10.1016/j.soilbio.2021.108422

[ref98] Wieder W. R. Bonan G. B. Allison S. D. (2013). Global soil carbon projections are improved by modelling microbial processes. Nat. Clim. Chang. 3, 909–912. doi: 10.1038/nclimate1951

[ref99] Wingeyer A. B. Walters D. T. Drijber R. A. Olk D. C. Arkebauer T. J. Verma S. B. . (2012). Fall conservation deep tillage stabilizes maize residues into soil organic matter. Soil Sci. Soc. Am. J. 76, 2154–2163. doi: 10.2136/sssaj2012.0121

[ref100] Xia L. L. Lam S. K. Wolf B. Kiese R. Chen D. Butterbach-Bahl K. (2018). Trade-offs between soil carbon sequestration and reactive nitrogen losses under straw return in global agroecosystems. Glob. Change Biol. 24, 5919–5932. doi: 10.1111/gcb.14466, 30295405

[ref101] Yang X. Ren W. Sun B. Zhang S. (2012). Effects of contrasting soil management regimes on total and labile soil organic carbon fractions in a loess soil in China. Geoderma 177, 49–56. doi: 10.1016/j.geoderma.2012.01.033

[ref102] Yu Y. Y. Turner N. C. Gong Y. H. Li F. M. Fang C. Ge L. J. . (2018). Benefits and limitations to straw-and plastic-film mulch on maize yield and water use efficiency: a meta-analysis across hydrothermal gradients. Eur. J. Agron. 99, 138–147. doi: 10.1016/j.eja.2018.07.005

[ref103] Zak D. R. Holmes W. E. White D. C. Peacock A. D. Tilman D. (2003). Plant diversity, soil microbial communities, and ecosystem function: are there any links? Ecology 84, 2042–2050. doi: 10.1890/02-0433

[ref104] Zechmeister-Boltenstern S. Keiblinger K. M. Mooshammer M. Peñuelas J. Richter A. Sardans J. . (2015). The application of ecological stoichiometry to plant–microbial–soil organic matter transformations. Ecol. Monogr. 85, 133–155. doi: 10.1890/14-0777.1

[ref105] Zhang P. Chen X. L. Wei T. Yang Z. Jia Z. K. Yang B. P. . (2016). Effects of straw incorporation on the soil nutrient contents, enzyme activities, and crop yield in a semiarid region of China. Soil Tillage Res. 160, 65–72. doi: 10.1016/j.still.2016.02.006

[ref106] Zhang M. Cheng G. Feng H. Sun B. H. Zhao Y. Chen H. X. . (2017). Effects of straw and biochar amendments on aggregate stability, soil organic carbon, and enzyme activities in the loess plateau, China. Environ. Sci. Pollut. Res. 24, 10108–10120. doi: 10.1007/s11356-017-8505-8, 28233202

[ref94] Zhang Y. X. Yang Q. L. Feng H. Chen J. Li Y. (2026). Meta-analysis shows the effects of straw return on soil organic carbon and total nitrogen in cropland. Chin. J. Eco-Agric. 34, 1–12. doi: 10.12357/cjea.20250505

[ref107] Zhao X. M. He L. Zhang Z. D. Wang H. B. Zhao L. P. (2016). Simulation of accumulation and mineralization (CO2 release) of organic carbon in chernozem under different straw return ways after corn harvesting. Soil Tillage Res. 156, 148–154. doi: 10.1016/j.still.2015.11.001

[ref108] Zhao S. Li K. Zhou W. Qiu S. Huang S. He P. (2016). Changes in soil microbial community, enzyme activities and organic matter fractions under long-term straw return in north-Central China. Agric. Ecosyst. Environ. 216, 82–88. doi: 10.1016/j.agee.2015.09.028

[ref109] Zheng Q. Hu Y. Zhang S. Noll L. Böckle T. Dietrich M. . (2019). Soil multifunctionality is affected by the soil environment and by microbial community composition and diversity. Soil Biol. Biochem. 136:107521. doi: 10.1016/j.soilbio.2019.107521, 31700196 PMC6837881

[ref110] Zhu L. Hu N. Zhang Z. Xu J. Tao B. Meng Y. (2015). Short-term responses of soil organic carbon and carbon pool management index to different annual straw return rates in a rice-wheat cropping system. Catena 135, 283–289. doi: 10.1016/j.catena.2015.08.008

[ref111] Zuo S. Wu D. Du Z. Xu C. Wu W. (2022). Effects of white-rot fungal pretreatment of corn straw return on greenhouse gas emissions from the North China plain soil. Sci. Total Environ. 807:150837. doi: 10.1016/j.scitotenv.2021.150837, 34627877

