# Supplementary material for: Keystone roles of carbon-degrading enzyme activities in mediating carbon in soils subjected to straw return: a global meta-analysis
Source: Front Microbiol. 2026 Feb 4;17:1739110. doi: 10.3389/fmicb.2026.1739110 (PMC12913515; doi:10.3389/fmicb.2026.1739110)
Supplement: Supplementary file 1 [file Supplementary_file_1.doc]

**Supplementary Materials**

**Brief description of this file**

**1. Supplementary figures**

**Fig. S1.** PRISMA diagram showing the process of locating publications included in this meta-analysis. PRISMA, Preferred Reporting Items for Systematic Reviews and Meta-analyses.

**Fig. S2.** Importance of predictors selected as potential parameters of Hy-EEAs from straw return, according to the random-meta-forest approach. Hy-EEAs, soil hydrolytic C-degrading extracellular enzyme activities; Clay, soil clay content (%); MAP, mean annual precipitation; Crop type, maize, rice, wheat, and other; Duration, experiment duration (year); MAT, mean annual temperature; Straw management, incorporated and surface; NDS, soluble NDS (% DM); Lignin, straw lignin content (% DM); LCI, lignocellulose index; Hem.+Cell., the sum of hemicellulos and cellulose; *lnR*-Soil pH, straw return-induced changes in soil pH; Fertilizer form, mixed, and urea.

**Fig. S3**. Dependence plots of straw return factors affecting Hy-EEAs. The figure shows the predicted effect of straw return (*y_i_*) as a function of the value of each variable in a random-forest meta-analysis. Little variation in *y_i_* across the values of a predictor shows the low predictive power of the predictor for *y_i_*. Hy-EEAs, soil hydrolytic C-degrading extracellular enzyme activities; Clay, soil clay content (%); MAP, mean annual precipitation; Crop type, maize, rice, wheat, and other; Duration, experiment duration (year); MAT, mean annual temperature; Straw management, incorporated and surface; NDS, soluble NDS (% DM); Lignin, straw lignin content (% DM); LCI, lignocellulose index; Hem.+Cell., the sum of hemicellulos and cellulose; *lnR*-Soil pH, straw return-induced changes in soil pH; Fertilizer form, mixed, and urea.

**Fig. S4.** Importance of predictors selected as potential parameters of MBC content from straw return, according to the random-meta-forest approach. MBC, soil microbial biomass carbon; Clay, soil clay content (%); Hy-EEAs, soil hydrolytic C-degrading extracellular enzyme activities; *lnR*-Hy-EEAs, straw return-induced changes in soil Hy-EEAs; MAP, mean annual precipitation; Duration, experiment duration (year); Crop type, maize, rice, wheat, and other; MAT, mean annual temperature; Straw management, incorporated and surface; Fertilizer form, mixed, and urea; Lignin, straw lignin content (% DM); NDS, soluble NDS (% DM); Hem.+Cell., the sum of hemicellulos and cellulose; LCI, lignocellulose index; Straw type, green plant biomass, mature aboveground biomass, senescent plant biomass, and straw.

**Fig. S5.** Dependence plots of straw return factors affecting MBC content. The figure shows the predicted effect of straw return (*y_i_*) as a function of the value of each variable in a random-forest meta-analysis. Little variation in *y_i_* across the values of a predictor shows the low predictive power of the predictor for *y_i_*. MBC, soil microbial biomass carbon; Clay, soil clay content (%); Hy-EEAs, soil hydrolytic C-degrading extracellular enzyme activities; *lnR*-Hy-EEAs, straw return-induced changes in soil Hy-EEAs; MAP, mean annual precipitation; Duration, experiment duration (year); Crop type, maize, rice, wheat, and other; MAT, mean annual temperature; Straw management, incorporated and surface; Fertilizer form, mixed, and urea; Lignin, straw lignin content (% DM); NDS, soluble NDS (% DM); Hem.+Cell., the sum of hemicellulos and cellulose; LCI, lignocellulose index; Straw type, green plant biomass, mature aboveground biomass, senescent plant biomass, and straw.

**Fig. S6.** Importance of predictors selected as potential parameters of SOC storage from straw return, according to the random-meta-forest approach. SOC, soil organic carbon; MAP, mean annual precipitation; Duration, experiment duration (year); Clay, soil clay content (%); MAT, mean annual temperature; Hy-EEAs, soil hydrolytic C-degrading extracellular enzyme activities; *lnR*-Hy-EEAs, straw return-induced changes in soil Hy-EEAs; Straw management, incorporated and surface; Hem.+Cell., the sum of hemicellulos and cellulose; NDS, soluble NDS (% DM); Crop type, maize, rice, wheat, and other; LCI, lignocellulose index; Lignin, straw lignin content (% DM); Fertilizer form, mixed, and urea; Straw type, green plant biomass, mature aboveground biomass, senescent plant biomass, and straw.

**Fig. S7.** Dependence plots of straw return factors affecting SOC storage. The figure shows the predicted effect of straw return (*y_i_*) as a function of the value of each variable in a random-forest meta-analysis. Little variation in *y_i_* across the values of a predictor shows the low predictive power of the predictor for *y_i_*. SOC, soil organic carbon; MAP, mean annual precipitation; Duration, experiment duration (year); Clay, soil clay content (%); MAT, mean annual temperature; Hy-EEAs, soil hydrolytic C-degrading extracellular enzyme activities; *lnR*-Hy-EEAs, straw return-induced changes in soil Hy-EEAs; Straw management, incorporated and surface; Hem.+Cell., the sum of hemicellulos and cellulose; NDS, soluble NDS (% DM); Crop type, maize, rice, wheat, and other; LCI, lignocellulose index; Lignin, straw lignin content (% DM); Fertilizer form, mixed, and urea; Straw type, green plant biomass, mature aboveground biomass, senescent plant biomass, and straw.

**Fig. S8.** Effects of straw return on Hy-EEAs for crop type, fertilizer form, and straw type. Hy-EEAs, soil hydrolytic C-degrading extracellular enzyme activities. Error bars represent 95% confidence intervals. The sample size for each variable is shown in the right column of the figure.

**Fig. S9.** Effects of straw return on soil MBC content for crop type, fertilizer form, starw type, and straw management. MBC, soil microbial biomass carbon. Error bars represent 95% confidence intervals. The sample size for each variable is shown in the right column of the figure.

**Fig. S10.** Effects of straw return on soil SOC storage for crop type, fertilizer form, starw type, and straw management. SOC, soil organic carbon. Error bars represent 95% confidence intervals. The sample size for each variable is shown in the right column of the figure.

**2. Supplementary tables**

**Table S1** Evaluation of model parameters used to explain soil C pool under straw return. Soil C pool includes soil dissolved organic C (DOC), easily oxidizable C (EOC), light fraction organic C (LFOC), microbial biomass C (MBC), particulate organic C (POC), and soil organic C (SOC).

**3. Supplementary materials and methods**

Soil hydrolytic C-degrading extracellular enzyme activities (Hy-EEAs)

**
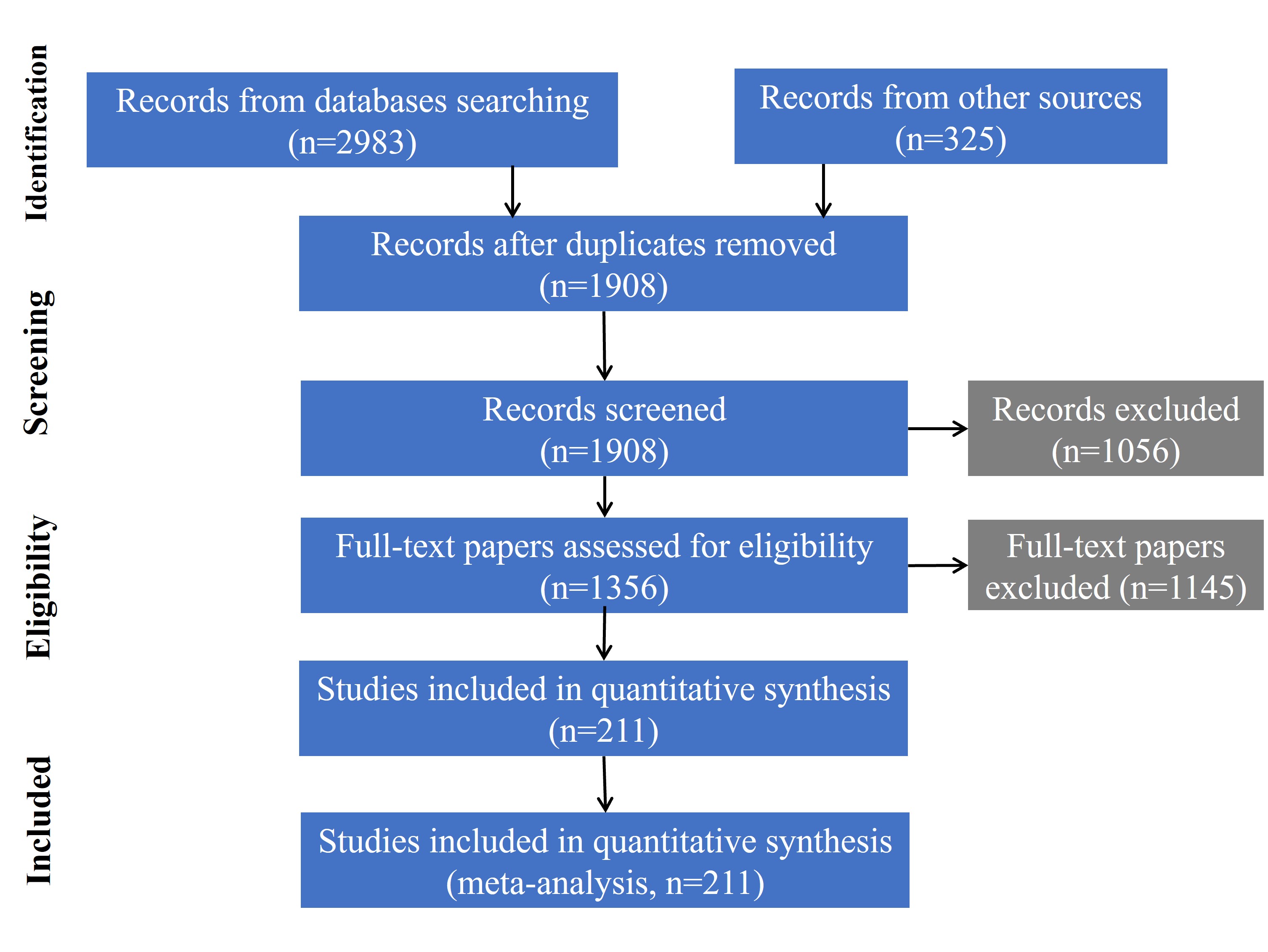
**

**Fig. S1.** PRISMA diagram showing the process of locating publications included in this meta-analysis. PRISMA,Preferred Reporting Items for Systematic Reviews and Meta-analyses.

**
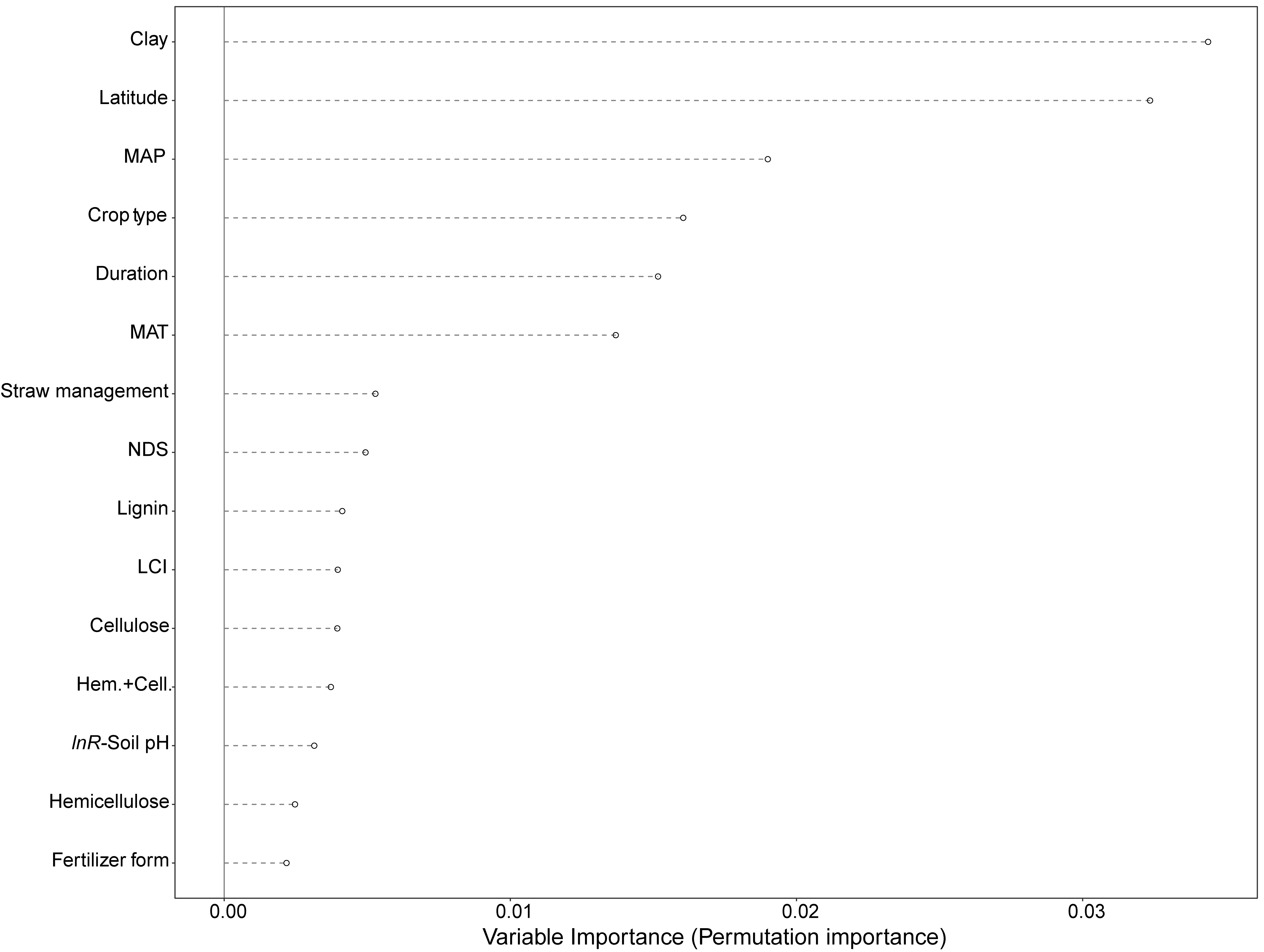
**

**Fig. S2.** Importance of predictors selected as potential parameters of Hy-EEAs from straw return, according to the random-meta-forest approach. Hy-EEAs, soil hydrolytic C-degrading extracellular enzyme activities; Clay, soil clay content (%); MAP, mean annual precipitation; Crop type, maize, rice, wheat, and other; Duration, experiment durantion (year); MAT, mean annual temperature; Straw management, incorporated and surface; NDS, soluble NDS (% DM); Lignin, straw lignin content (% DM); LCI, lignocellulose index; Hem.+Cell., the sum of hemicellulos and cellulose; *lnR*-Soil pH, straw return-induced changes in soil pH; Fertilizer form, mixed, and urea.

**
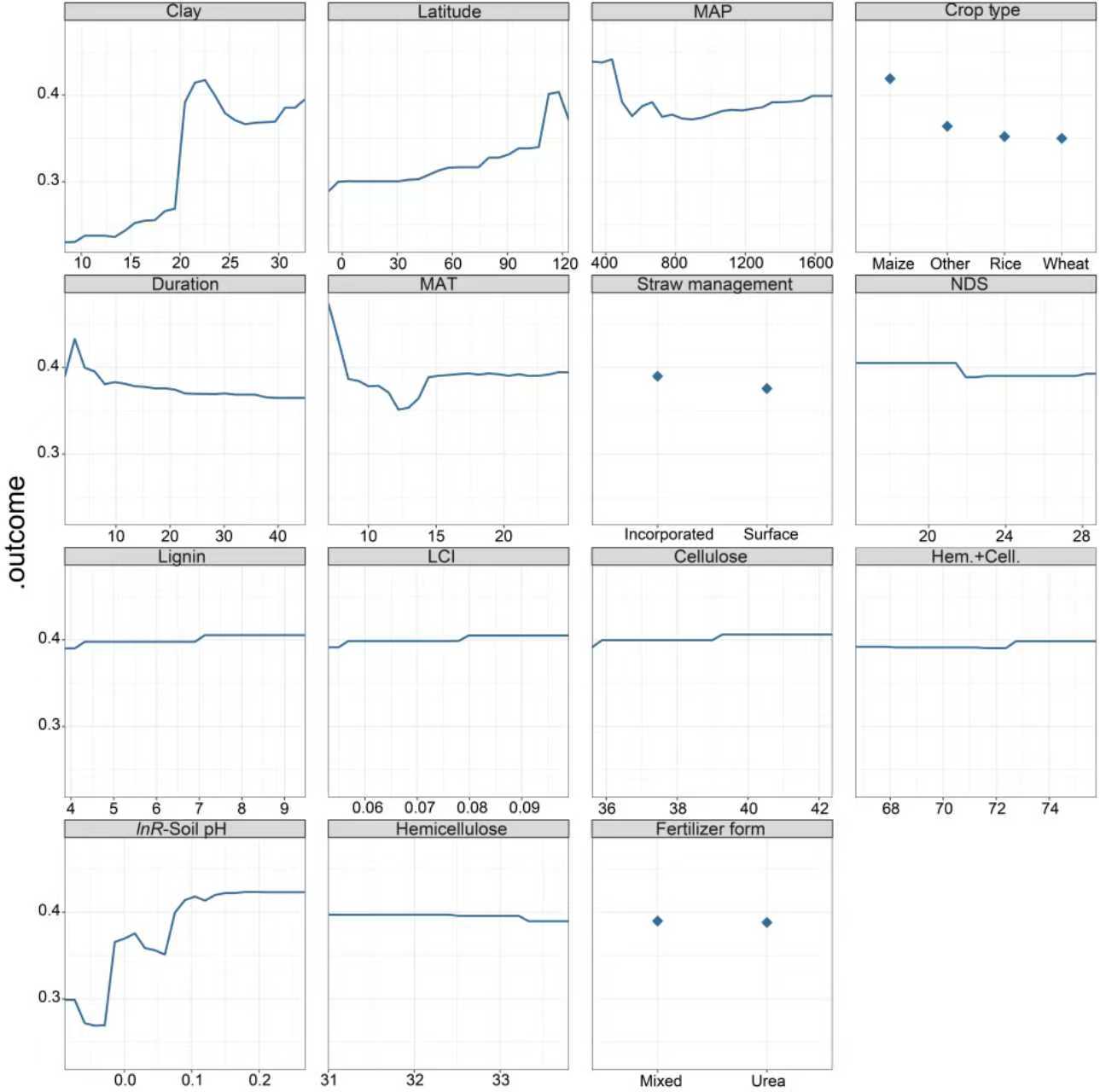
**

**Fig. S3**. Dependence plots of straw return factors affecting Hy-EEAs. The figure shows the predicted effect of straw return (*y_i_*) as a function of the value of each variable in a random-forest meta-analysis. Little variation in *y_i_* across the values of a predictor shows the low predictive power of the predictor for *y_i._* Hy-EEAs, soil hydrolytic C-degrading extracellular enzyme activities; Clay, soil clay content (%); MAP, mean annual precipitation; Crop type, maize, rice, wheat, and other; Duration, experiment durantion (year); MAT, mean annual temperature; Straw management, incorporated and surface; NDS, soluble NDS (% DM); Lignin, straw lignin content (% DM); LCI, lignocellulose index; Hem.+Cell., the sum of hemicellulos and cellulose; *lnR*-Soil pH, straw return-induced changes in soil pH; Fertilizer form, mixed, and urea.


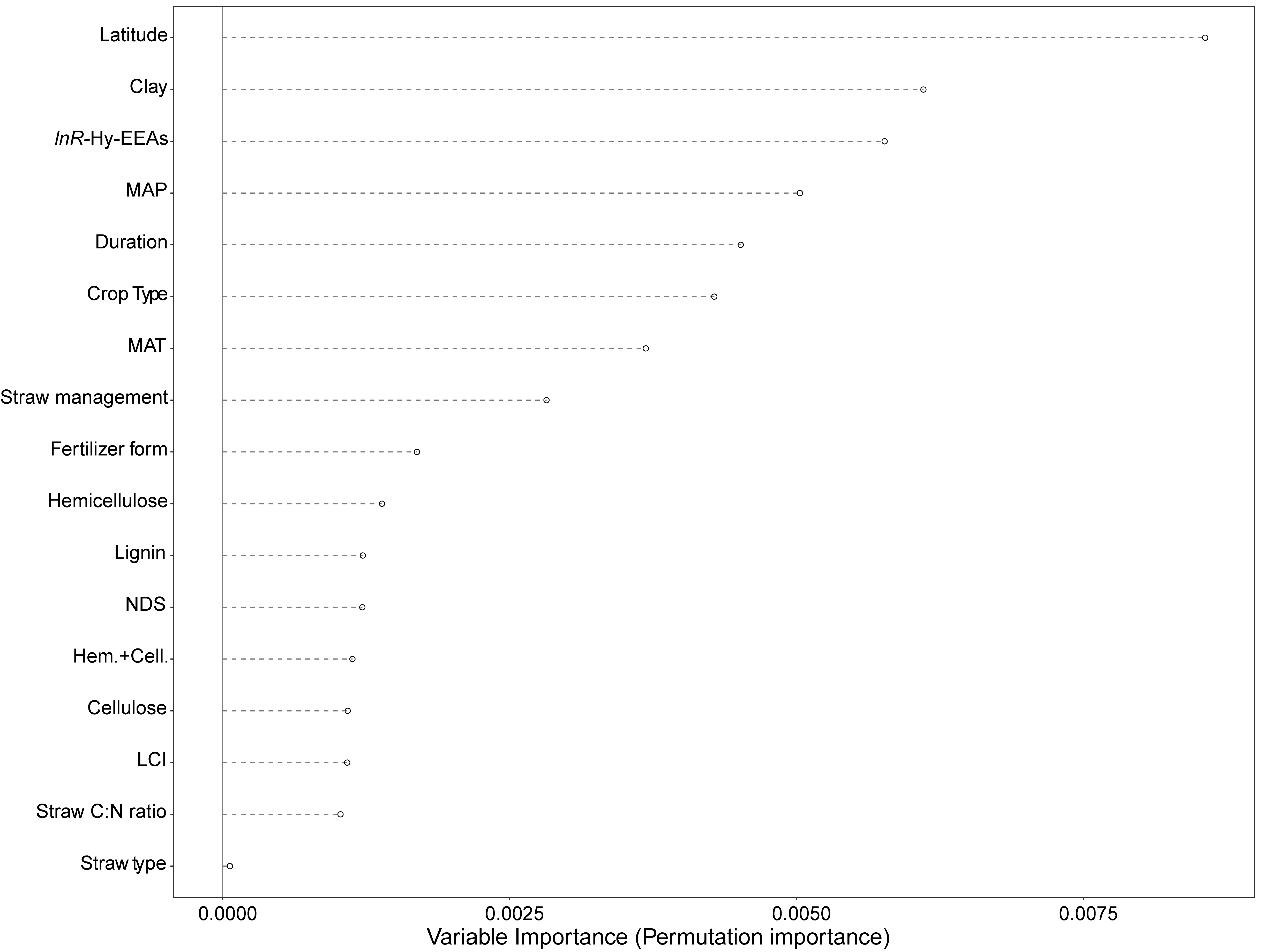


**Fig. S4.** Importance of predictors selected as potential parameters of MBC content from straw return, according to the random-meta-forest approach. MBC, soil microbial biomass carbon; Clay, soil clay content (%); Hy-EEAs, soil hydrolytic C-degrading extracellular enzyme activities; *lnR*-Hy-EEAs, straw return-induced changes in soil Hy-EEAs; MAP, mean annual precipitation; Duration, experiment duration (year); Crop type, maize, rice, wheat, and other; MAT, mean annual temperature; Straw management, incorporated and surface; Fertilizer form, mixed, and urea; Lignin, straw lignin content (% DM); NDS, soluble NDS (% DM); Hem.+Cell., the sum of hemicellulos and cellulose; LCI, lignocellulose index; Straw type, green plant biomass, mature aboveground biomass, senescent plant biomass, and straw.


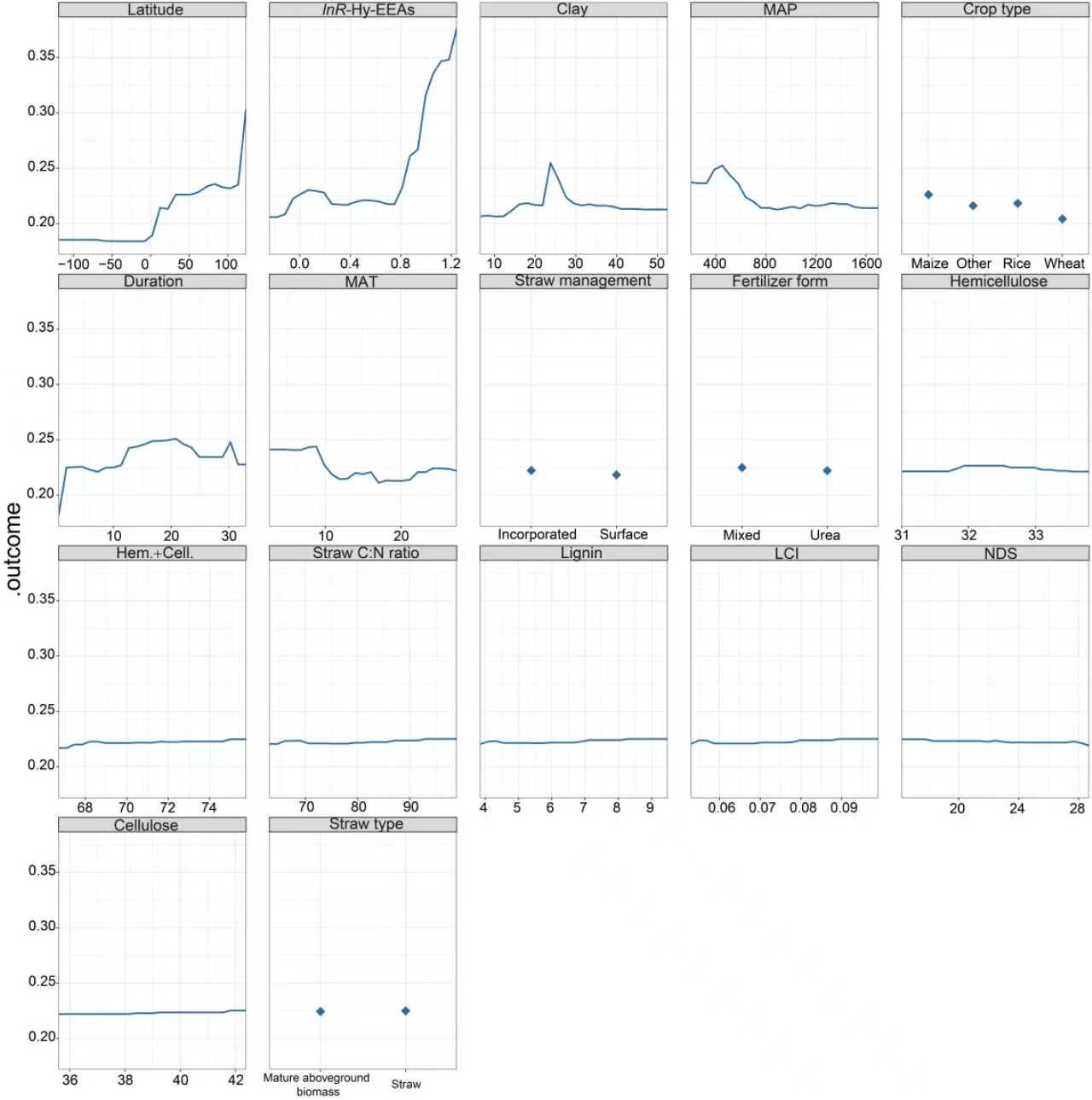


**Fig. S5** Dependence plots of straw return factors affecting MBC content. The figure shows the predicted effect of straw return (*y_i_*) as a function of the value of each variable in a random-forest meta-analysis. Little variation in *y_i_* across the values of a predictor shows the low predictive power of the predictor for *y_i_*. MBC, soil microbial biomass carbon; Clay, soil clay content (%); Hy-EEAs, soil hydrolytic C-degrading extracellular enzyme activities; *lnR*-Hy-EEAs, straw return-induced changes in soil Hy-EEAs; MAP, mean annual precipitation; Duration, experiment duration (year); Crop type, maize, rice, wheat, and other; MAT, mean annual temperature; Straw management, incorporated and surface; Fertilizer form, mixed, and urea; Lignin, straw lignin content (% DM); NDS, soluble NDS (% DM); Hem.+Cell., the sum of hemicellulos and cellulose; LCI, lignocellulose index; Straw type, green plant biomass, mature aboveground biomass, senescent plant biomass, and straw.


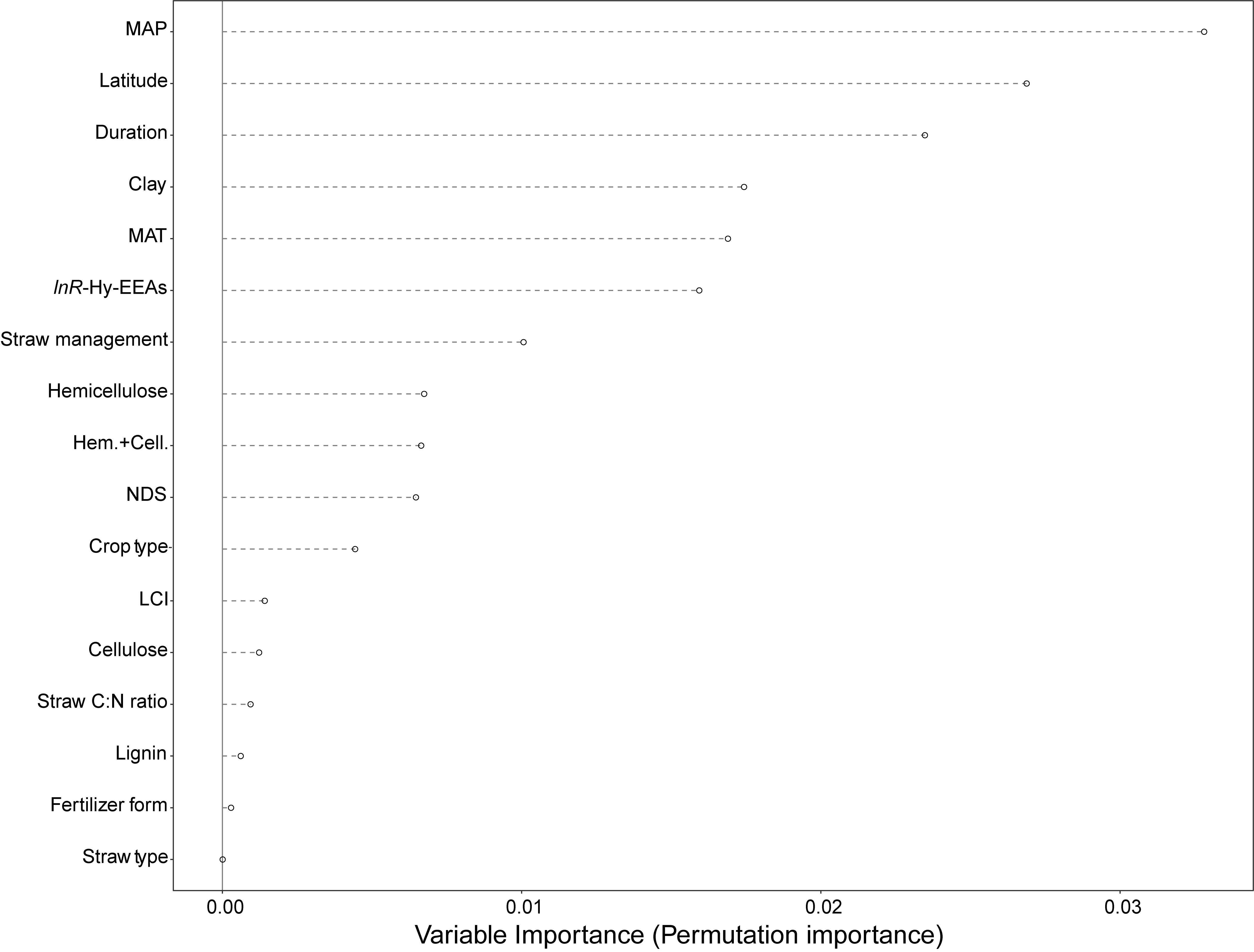


**Fig. S6.** Importance of predictors selected as potential parameters of SOC storage from straw return, according to the random-meta-forest approach. SOC, soil organic carbon; MAP, mean annual precipitation; Duration, experiment duration (year); Clay, soil clay content (%); MAT, mean annual temperature; Hy-EEAs, soil hydrolytic C-degrading extracellular enzyme activities; *lnR*-Hy-EEAs, straw return-induced changes in soil Hy-EEAs; Straw management, incorporated and surface; Hem.+Cell., the sum of hemicellulos and cellulose; NDS, soluble NDS (% DM); Crop type, maize, rice, wheat, and other; LCI, lignocellulose index; Lignin, straw lignin content (% DM); Fertilizer form, mixed, and urea; Straw type, green plant biomass, mature aboveground biomass, senescent plant biomass, and straw.

**

**

**Fig. S7.** Dependence plots of straw return factors affecting SOC storage. The figure shows the predicted effect of straw return (*y_i_*) as a function of the value of each variable in a random-forest meta-analysis. Little variation in *y_i_* across the values of a predictor shows the low predictive power of the predictor for *y_i_.* SOC, soil organic carbon; MAP, mean annual precipitation; Duration, experiment duration (year); Clay, soil clay content (%); MAT, mean annual temperature; Hy-EEAs, soil hydrolytic C-degrading extracellular enzyme activities; *lnR*-Hy-EEAs, straw return-induced changes in soil Hy-EEAs; Straw management, incorporated and surface; Hem.+Cell., the sum of hemicellulos and cellulose; NDS, soluble NDS (% DM); Crop type, maize, rice, wheat, and other; LCI, lignocellulose index; Lignin, straw lignin, content (% DM); Fertilizer form, mixed, and urea; Straw type, green plant biomass, mature aboveground biomass, senescent plant biomass, and straw.

**
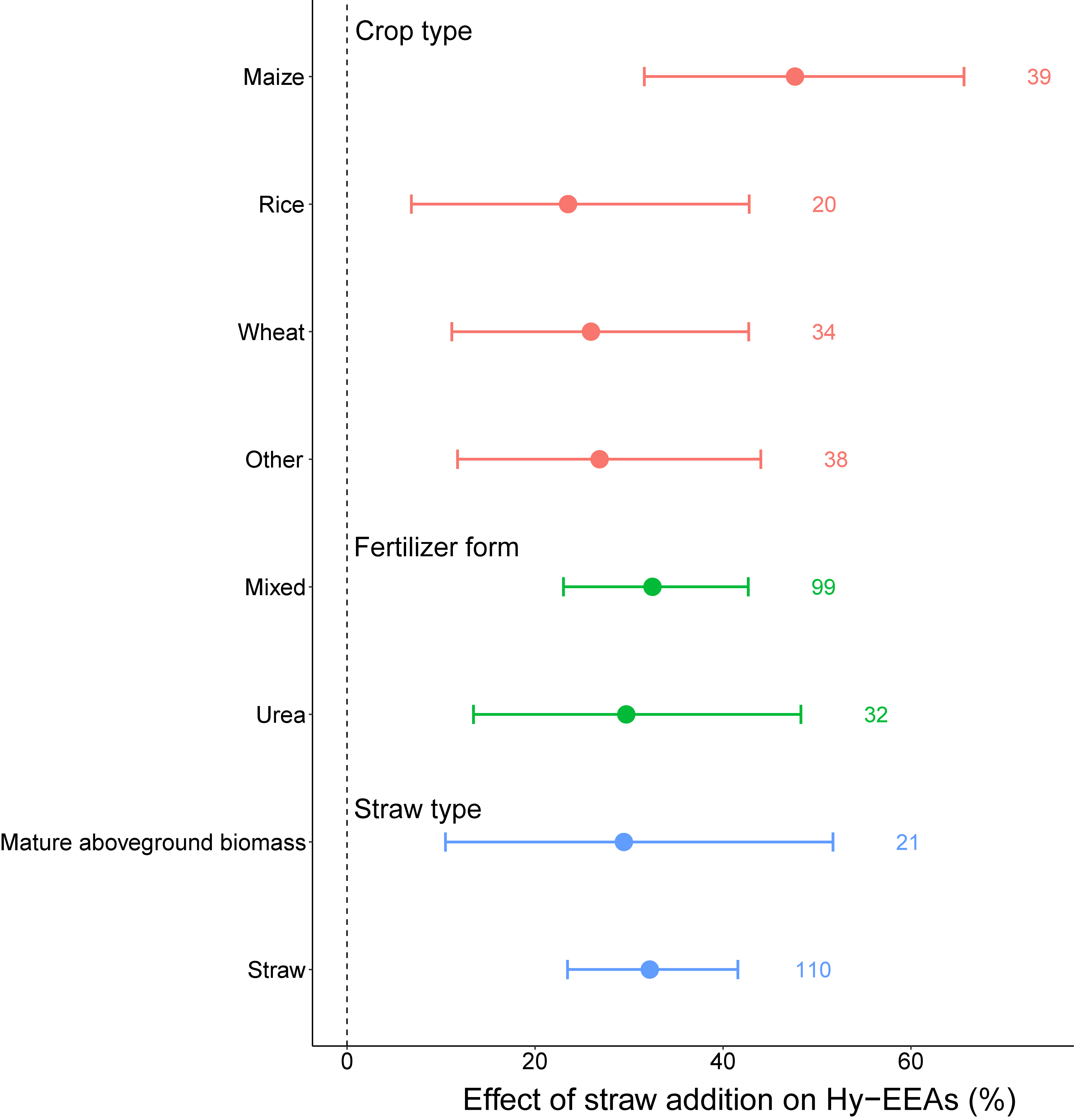
**

**Fig. S8.** Effects of straw return on Hy-EEAs for crop type, fertilizer form, and straw type. Hy-EEAs, soil hydrolytic C-degrading extracellular enzyme activities. Error bars represent 95% confidence intervals. The sample size for each variable is shown in the right column of the figure.


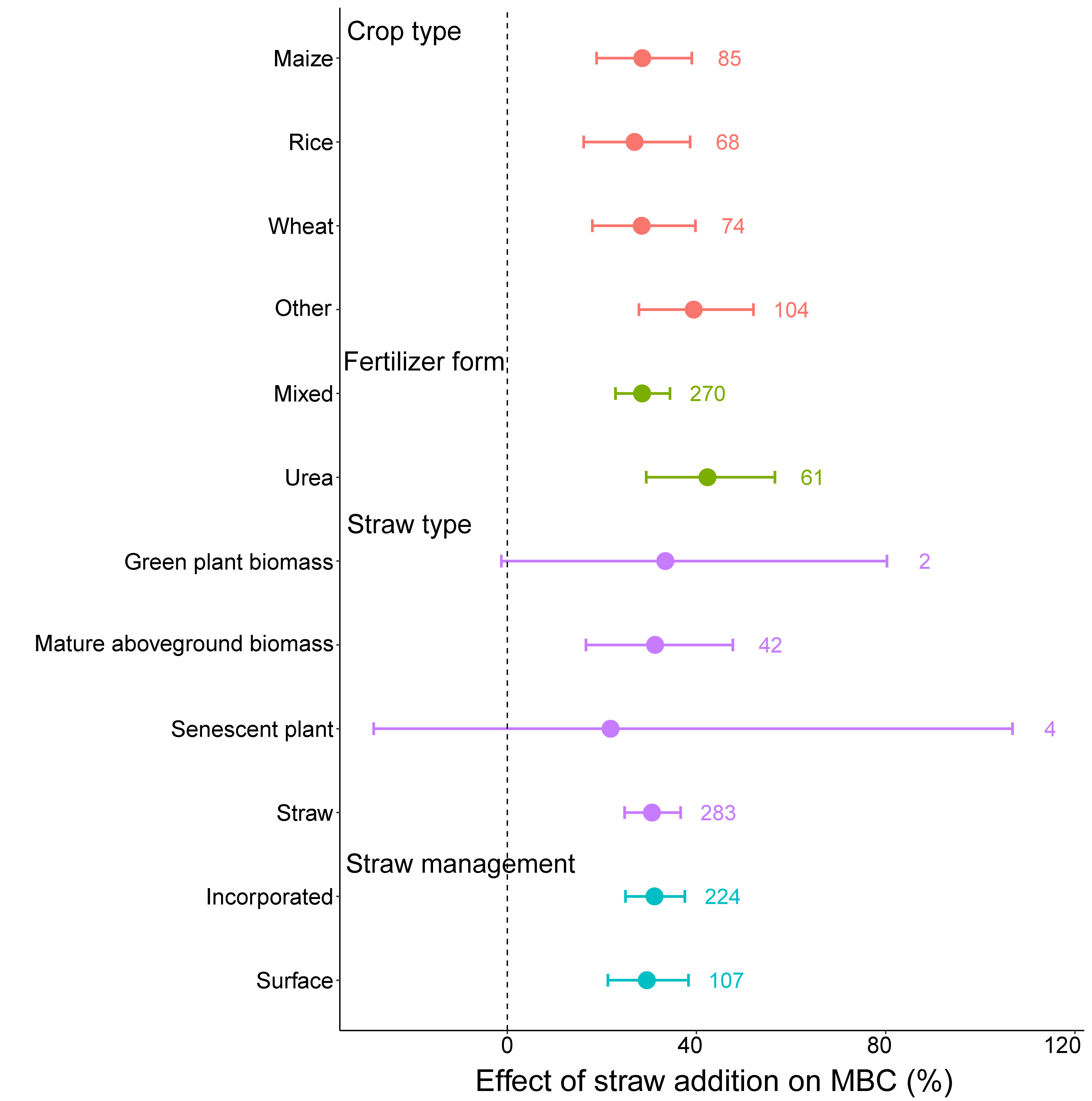


**Fig. S9.** Effects of straw return on soil MBC content for crop type, fertilizer form, starw type, and straw management. MBC, soil microbial biomass carbon. Error bars represent 95% confidence intervals. The sample size for each variable is shown in the right column of the figure.


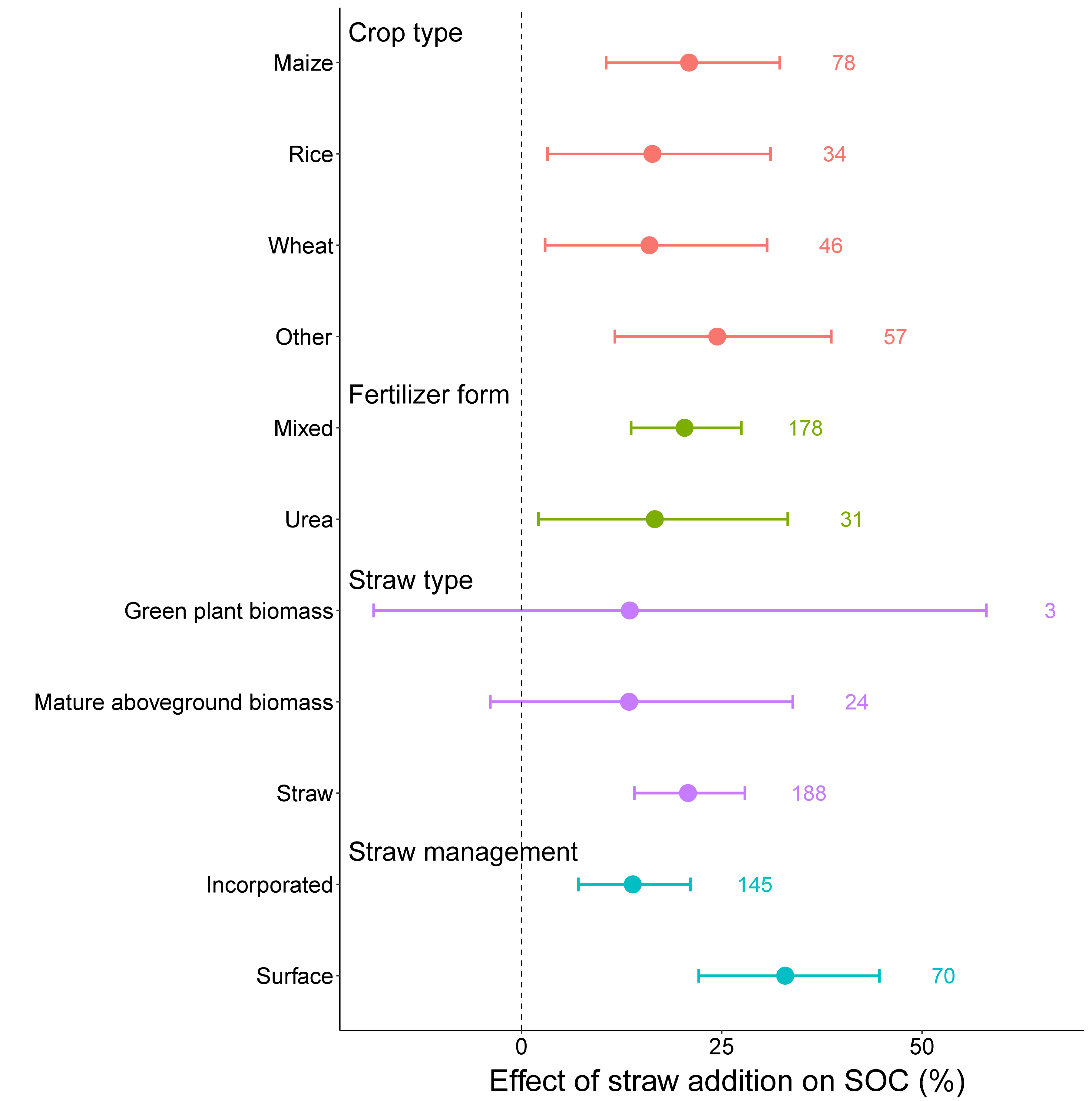


**Fig. S10.** Effects of straw return on soil SOC storage for crop type, fertilizer form, starw type, and straw management. SOC, soil organic carbon. Error bars represent 95% confidence intervals. The sample size for each variable is shown in the right column of the figure.

**2. Supplementary tables**

**Table S1** Evaluation of model parameters used to explain soil C pool (DOC, EOC, MBC, LFOC, SOC, and POC) under straw return.

| Soil carbon pools | Variable | F | *R^2^* | SE | t | *df* | *p* | *n* |
| --- | --- | --- | --- | --- | --- | --- | --- | --- |
| DOC | Latitude | 1.026 | 0.002 | 0.006 | -1.013 | 98 | 0.314 | 101 |
|  | **Elevation** | **5.184** | **0.075** | **0.000** | **2.277** | **99** | **0.025** | **101** |
|  | Duration | 0.055 | 0.002 | 0.063 | -0.235 | 99 | 0.815 | 101 |
|  | MAT | 0.116 | 0.032 | 0.007 | 0.341 | 99 | 0.734 | 101 |
|  | MAP | 0.018 | 0.042 | 0.000 | -0.135 | 99 | 0.893 | 101 |
|  | Clay | 0.633 | 0.014 | 0.004 | 0.796 | 99 | 0.428 | 101 |
|  | pH | 0.289 | 0.041 | 0.027 | -0.537 | 99 | 0.592 | 101 |
|  | Soil C:N ratios | 0.011 | 0.003 | 0.003 | -0.104 | 99 | 0.917 | 101 |
| EOC | Latitude | 24.236 | 0.002 | 0.007 | 4.923 | 45 | <.0001 | 47 |
|  | Elevation | 1.729 | 0.093 | 0.000 | 1.315 | 45 | 0.195 | 47 |
|  | Duration | 3.368 | 0.045 | 0.107 | -1.835 | 45 | 0.073 | 47 |
|  | **MAT** | **17.624** | **0.266** | **0.010** | **-4.198** | **45** | **0.001** | **47** |
|  | **MAP** | **17.641** | **0.306** | **0.000** | **-4.200** | **45** | **0.001** | **47** |
|  | Clay | 0.155 | 0.002 | 0.005 | 0.394 | 45 | 0.696 | 47 |
|  | pH | 0.281 | 0.149 | 0.035 | 0.530 | 45 | 0.599 | 47 |
|  | Soil C:N ratios | 0.089 | 0.008 | 0.009 | -0.299 | 45 | 0.767 | 47 |
| MBC | Latitude | 1.713 | 0.001 | 0.002 | 1.309 | 328 | 0.192 | 330 |
|  | Elevation | 0.506 | 0.002 | 0.000 | 0.711 | 329 | 0.477 | 330 |
|  | Duration | 2.295 | 0.014 | 0.043 | -1.515 | 329 | 0.131 | 330 |
|  | MAT | 0.609 | 0.004 | 0.004 | -0.780 | 329 | 0.436 | 330 |
|  | MAP | 3.484 | 0.002 | 0.000 | -1.867 | 329 | 0.063 | 330 |
|  | Clay | 2.041 | 0.001 | 0.002 | -1.429 | 329 | 0.154 | 330 |
|  | pH | 3.795 | 0.011 | 0.018 | 1.948 | 329 | 0.052 | 330 |
|  | Soil C:N ratios | 1.898 | 0.018 | 0.001 | 1.378 | 329 | 0.169 | 330 |
| LFOC | Latitude | 3.026 | 0.002 | 0.017 | 1.740 | 35 | 0.091 | 37 |
|  | Elevation | 1.337 | 0.001 | 0.000 | 1.156 | 35 | 0.255 | 37 |
|  | Duration | 1.105 | 0.023 | 0.244 | -1.051 | 35 | 0.300 | 37 |
|  | **MAT** | **6.389** | **0.127** | **0.023** | **-2.528** | **35** | **0.016** | **37** |
|  | MAP | 3.659 | 0.074 | 0.000 | -1.913 | 35 | 0.064 | 37 |
|  | Clay | 1.890 | 0.123 | 0.009 | 1.375 | 35 | 0.178 | 37 |
|  | pH | 0.947 | 0.032 | 0.074 | 0.973 | 35 | 0.337 | 37 |
|  | Soil C:N ratios | 1.908 | 0.025 | 0.019 | -1.381 | 35 | 0.176 | 37 |
| SOC | Latitude | 0.130 | 0.001 | 0.003 | 0.361 | 213 | 0.718 | 215 |
|  | Elevation | 0.018 | 0.000 | 0.000 | 0.135 | 214 | 0.893 | 215 |
|  | Duration | 1.091 | 0.006 | 0.054 | -1.045 | 214 | 0.297 | 215 |
|  | MAT | 0.005 | 0.002 | 0.005 | 0.067 | 214 | 0.947 | 215 |
|  | MAP | 0.843 | 0.009 | 0.000 | -0.918 | 214 | 0.360 | 215 |
|  | Clay | 0.209 | 0.000 | 0.003 | -0.457 | 214 | 0.648 | 215 |
|  | pH | 2.536 | 0.027 | 0.023 | 1.592 | 214 | 0.113 | 215 |
|  | Soil C:N ratios | 0.273 | 0.006 | 0.001 | 0.523 | 214 | 0.602 | 215 |
| POC | Latitude | 0.061 | 0.005 | 0.005 | 0.246 | 30 | 0.807 | 32 |
|  | Elevation | 1.301 | 0.039 | 0.000 | 1.141 | 30 | 0.263 | 32 |
|  | Duration | 1.885 | 0.001 | 0.087 | 1.373 | 30 | 0.180 | 32 |
|  | **MAT** | **7.073** | **0.216** | **0.008** | **-2.660** | **30** | **0.012** | **32** |
|  | MAP | 3.581 | 0.084 | 0.000 | -1.892 | 30 | 0.068 | 32 |
|  | Clay | 6.670 | 0.224 | 0.004 | 2.583 | 30 | 0.015 | 32 |
|  | pH | 0.059 | 0.001 | 0.032 | -0.242 | 30 | 0.810 | 32 |
|  | Soil C:N ratios | 0.578 | 0.070 | 0.008 | 0.760 | 30 | 0.453 | 32 |

Model predictors with significant correlation (*p* < 0.05) showed in bold. *df,* denominator degree of freedom. DOC, soil dissolved organic C; EOC, easily oxidizable C; LFOC, light fraction organic C; MBC, microbial biomass C; POC, particulate organic C; SOC, soil organic C. Clay, soil clay content in %; MAT, mean annual temperature; MAP, mean annual precipitation; Soil C: N ratios, soil organic carbon: soil total nitrogen. *lnR*, log-transformed response ratio.

**3. Supplementary materials and methods**

**Soil hydrolytic C-degrading extracellular enzyme activities**

Soil hydrolytic C-degrading extracellular enzyme activities (Hy*-*EEAs) include AG, BG, BX, and CBH (Li et al., 2025). The overall responses of Hy-EEAs were calculated from the sum of their components when two, three, or four kinds of Hy-EEAs were reported in the same article. We used the sum of Hy-EEAs as the proxy (Jian et al., 2016), calculated as follows:

$Hy-EEAs=AG+BG+BX+CBX$ (1)

The standard deviation (*SD)* for Hy-EEAs in the control and straw return treatments was calculated as follows (Chave et al., 2004):

$SD_{X}=\sqrt{(SD_{AG})^{2}+(SD_{BG})^{2}+(SD_{BX})^{2}+(SD_{CBH})^{2}}$ (2)

where *SD_X_* is the newly calculated standard deviations, and *SD_AG_*, *SD_BG_*, *SD_BX_*, and *SD_CBH_* are standard deviations of individual enzymes.

Reference cited:

Chave, J., Condit, R., Aguilar, S., Hernandez, A., Lao, S., Perez, R., 2004. Error propagation and scaling for tropical forest biomass estimates. Philosophical Transactions of the Royal Society B: Biological Sciences, 359, 409‒420.

<https://doi.org/10.1098/rstb.2003.1425>

Jian, S., Li, J., Chen, J., Wang, G., Mayes, M. A., Dzantor, K. E., 2016. Soil extracellular enzyme activities, soil carbon and nitrogen storage under nitrogen fertilization: A meta-analysis. Soil Biology and Biochemistry, 101, 32‒43. <http://dx.doi.org/10.1016/j.soilbio.2016.07.003>

Li, Y., Lu, J., Feng, H. et al. 2025. Soil cellulase activity responds to straw return and correlates with soil organic carbon dynamics: A global meta-analysis. Plant Soil. https://doi.org/10.1007/s11104-025-08223-7
